# Causal Relationship between Gut Microbiota and Gout: A Two-Sample Mendelian Randomization Study

**DOI:** 10.3390/nu15194260

**Published:** 2023-10-05

**Authors:** Mengna Wang, Jiayao Fan, Zhaohui Huang, Dan Zhou, Xue Wang

**Affiliations:** 1 Wuxi School of Medicine, Jiangnan University, Wuxi 214122, China; 2 School of Public Health and the Second Affiliated Hospital, Zhejiang University School of Medicine, Hangzhou 310058, China; 3 Affiliated Hospital of Jiangnan University, Wuxi 214062, China

**Keywords:** gout, gut microbiota, phylum *Actinobacteria*, Mendelian Randomization, urate

## Abstract

Gout is a form of prevalent and painful inflammatory arthritis characterized by elevated serum urate (SUA) levels. The gut microbiota (GM) is believed to influence the development of gout and SUA levels. Our study aimed to explore the causal relationship between GM composition and gout, as well as SUA levels, utilizing a two-sample Mendelian Randomization (MR) approach. A total of 196 GM taxa from five levels were available for analysis. We identified five taxa associated with SUA levels and 10 taxa associated with gout. In reverse MR analysis, we discovered that gout affected the composition of five GM taxa, while SUA levels influenced the composition of 30 GM taxa. Combining existing research, our study unveiled a potential negative feedback loop between phylum *Actinobacteria* and SUA levels, establishing connections with gout. We also proposed two novel associations connecting GM taxa (genus *Faecalibacterium* and genus *Prevotella9*), SUA levels, and gout. These findings provide compelling evidence of causal relationships between specific GM taxa with SUA levels and gout, contributing valuable insights for the treatment of gout.

## 1. Introduction

Gout, as one of the most common arthritic diseases, is a recurrent inflammatory disease caused by the deposition of uric acid crystals in the joint synovium, capsule, cartilage, kidneys, and other tissues [1]. The global prevalence of gout has been steadily increasing since the 20th century [2,3,4,5,6,7,8]. This trend is likely due to lifestyle modifications, such as the adoption of diets rich in purine and increased alcohol intake [9]. Gout attacks cause varying degrees of pain, leading to limited movement, difficulty in walking, and even a fear of physical contact, which reduces patients’ quality of life [10]. Furthermore, gout is frequently associated with other metabolic disorders that lead to serious complications [11]. Gout has become a hot public health problem. It is crucial to understand the modifiable risk factors and potential consequences of gout.

The human gut microbiota (GM) consists of a diverse range of bacteria, with approximately 1000–1150 different bacterial species that play a vital role in maintaining overall health [12]. The GM is in a state of dynamic development and can easily be disrupted due to the balance of environmental factors, leading to the onset of various diseases [13], such as depression [14] and Alzheimer’s disease (AD) [15]. The relationships between diseases and traits with GM are typically bidirectional [16,17]. A study indicated that phytoestrogens could influence the composition of GM, increasing the abundance of probiotic bacteria, which, in turn, can improve breast cancer patients’ survival [18]. Similarly, numerous studies have examined the relationship between GM and gout. Individuals with gout have a distinct GM composition compared to healthy volunteers [19,20,21]. GM may influence the metabolism of purines and short-chain fatty acids; ultimately, they will affect uric acid metabolism. Although these studies have confirmed a GM imbalance in gout patients, the causal relationship between GM dysbiosis and the development of gout remains unclear. Therefore, further research is necessary to investigate the causal association between gout and GM.

Mendelian randomization (MR) leverages genetic variations as instrumental variables (IVs) to assess the causal relationships between exposures and outcomes [22]. Significant advancements in MR methodology have led to improved power and interpretability in recent years [23]. In our study, we utilized a two-sample MR approach to estimate the causal relationship between GM and gout. On the other hand, serum urate (SUA) levels are an important indicator of gout. Previous research has indicated that approximately 30% of uric acid is excreted through the intestines, and one study pointed out that GM can serve as a potential target for controlling the mechanisms underlying hyperuricemia [24]. This suggests an association between GM and SUA levels, but there is currently limited research to establish a causal relationship between GM and SUA levels. Therefore, MR analysis was also conducted on GM and SUA in our study. To investigate the potential causal impact of gout and SUA levels on the GM, we also performed a reverse MR analysis using gout-related single nucleotide polymorphisms (SNPs) and SUA-related SNPs as IVs, with gout and SUA levels as the exposure and GM as the outcome.

## 2. Materials and Methods

### 2.1. Ethics Statement

**Figure 1:**
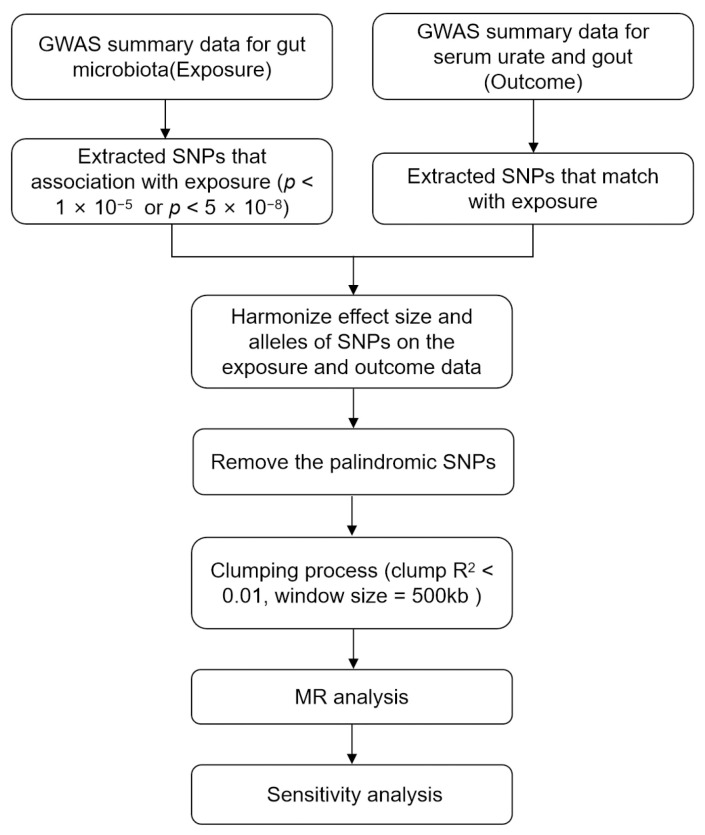
The flowchart of the study. GWAS, genome wide association study; SNPs, single nucleotide polymorphisms; MR, Mendelian randomization.

We utilized published genome wide association study (GWAS) summary statistics in our study. No new data collection or additional ethical approvals were required for this study. Figure 1 illustrates the flowchart of the study. IVs were chosen by applying rigorous inclusion and exclusion criteria to replace the GM. This analysis adhered to the STROBE-MR guidelines for reporting MR results [25], and it followed the three fundamental assumptions of MR (Table S1) [26].

### 2.2. Gut Microbiota Sample

Our study utilized GWAS summary statistics data of GM from the MiBioGen [27] consortium, which analyzed 16s ribosomal RNA gene sequencing profiles and genotyping data from 18,340 individuals across 24 cohorts (Table S2). This analysis encompassed a total of 211 taxa, which consisted of 131 genera, 35 families, 20 orders, 16 classes, and 9 phyla. For more details, see study [28].

### 2.3. Serum Urate Levels and Gout Samples

The summary statistics for SUA levels were derived from a trans-ethnic meta-analysis of GWAS, publicly accessible, comprising data from 457,690 individuals across 74 studies. GWAS data on gout were extracted from a trans-ethnic meta-analysis comprising 763,813 participants with 13,179 gout cases in the same study (Table S2). For more details, see the original article [29].

### 2.4. Selection of Instrumental Variables

To ensure data robustness and the accuracy of results, SNPs associated with GM taxa reached a genome-wide statistical significance threshold (*p* < 5 × 10^−8^) [30]. In addition, we also selected SNPs associated with each GM taxon at a relatively comprehensive threshold (*p* < 1.0 × 10^−5^) as candidate IVs in accordance with a previous study [31]. Next, we performed a linkage disequilibrium (LD) analysis clumping SNPs, and the LD threshold was set to r^2^ < 0.01, and the window size was set to 500 kb. The LD was estimated using a reference panel from the European 1000 Genomes Projects. Simultaneously, ambiguous, duplicate, and palindromic SNPs were removed. Finally, we assessed the strength of IVs by calculating the *F*-statistic. There was no strong evidence of a weak instrument bias if the *F*-statistic was ≥10 [32].

### 2.5. Mendelian Randomization Analysis

We performed bidirectional two-sample MR analyses to investigate the causal relationship of GM with SUA levels and gout. If a particular taxon had only one SNP as an IV, the MR analysis used the Wald ratio method for estimation. For taxa with multiple IVs, other methods were employed, including the inverse-variance-weighted test (IVW) [33], the weighted median (WM) method [34], the MR-Egger regression test [35], and Weighted mode [36]. A significance level of *p* < 0.05 was considered to determine the statistical significance and evidence for potential causal effects [37]. In this study, we utilized the Bonferroni method for multiple-testing correction.

### 2.6. Sensitivity Analysis

To evaluate the robustness of the results, we employed Cochrane’s Q test to detect heterogeneity among the IVs [33,34]. We utilized the intercept of MR-Egger regression to assess the potential presence of horizontal pleiotropy [35]. We considered a significance level of *p* < 0.05 to determine statistical significance. Additionally, a leave-one-out analysis was conducted to evaluate whether a single SNP was driving the significant result.

All statistical analyses were conducted using R (version 4.2.2). The IVW, WM, MR-Egger regression method, and leave-one-out analysis were performed using the “Two Sample MR” package (version 0.5.6) [38].

## 3. Results

### 3.1. Selection of Instrumental Variables

We excluded 15 unknown GM taxa from the MiBioGen consortium study, resulting in a total of 196 GM taxa that were included in subsequent MR analyses. We performed the quality control of SNPs according to the process outlined in Figure 1. A total of 14,575 SNPs associated with GM passed the locus-wide significance threshold of *p* < 1 × 10^−5^. Subsequently, harmonization and clumping procedures were applied, leading to the removal of palindrome SNPs and a reduction in the impact of LD. Ultimately, the number of IVs in relation to the 196 GM taxa for SUA and gout became 2410 and 2412, respectively. The *F*-statistics of IVs ranged from 11.03 to 206.84, all of which were notably greater than 10, indicating the absence of weak instrument bias (Tables S3 and S4).

At the other level of *p* < 5 × 10^−8^, a total of 1394 SNPs associated with GM were selected. After harmonization and clumping, 28 SNPs were associated with 20 taxa for gout and 29 SNPs were associated with 21 taxa for SUA. When considering the 196 GM taxa as a whole, 12 SNPs associated with the entire GM were selected as IVs. Each SNP demonstrated adequate validity, with all *F*-statistics exceeding 10 (Table S5).

In the reverse MR analysis, applying the same quality control criteria as in the previous analysis, we identified SNPs associated with gout and SUA at the level of *p* < 5 × 10^−8^. Ultimately, we identified 6 SNPs associated with gout for 5 taxa and 35 SNPs associated with SUA for 31 taxa. Moreover, when considering the 196 GM taxa as a whole, we selected 28 SNPs associated with SUA and 6 SNPs associated with gout as IVs with a level of *p* < 5 × 10^−8^. Each SNP demonstrated adequate validity, as all *F*-statistics exceeded 10 (Table S6).

### 3.2. Results of MR Analysis (Locus-Wide Significance, p < 1 × 10^−5^)

#### 3.2.1. Causal Effects of GM on SUA

**Figure 2:**
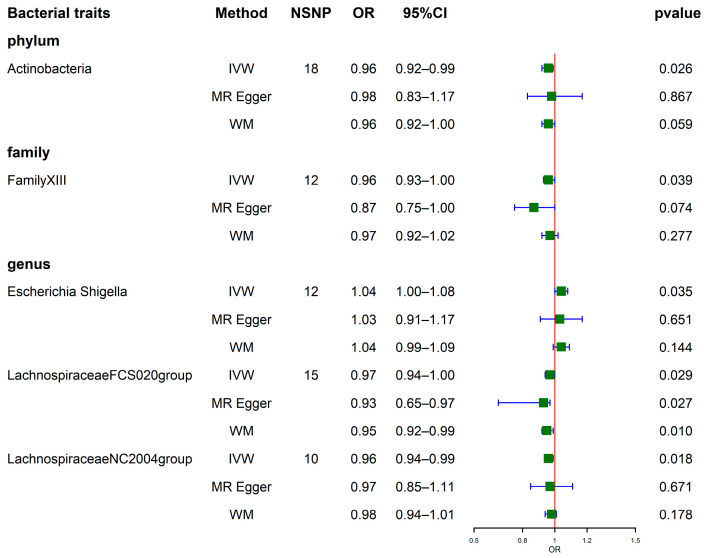
Forest plots show causal-effect estimates of GM with SUA (locus-wide significance, *p* < 1 × 10^−5^). IVW, Inverse-variance weighted; WM, Weighted median; NSNP, the number of SNP; OR, odds ratio; CI, confidence interval.

Among the results, we identified 1 phylum, 1 family, and 3 genera that were associated with SUA levels. Regarding the phylum and family levels, the MR estimates of IVW indicated that *Actinobacteria* (odds ratio (OR) = 0.96, 95% confidence interval (CI), 0.92–0.99, *p* = 0.026) and family XIII (OR = 0.96, 95% CI, 0.93–1.00, *p* = 0.039) were negatively correlated with SUA levels. Regarding the genus level, IVW analyses revealed that *Lachnospiraceae FCS020* group (OR = 0.97, 95% CI, 0.94–1.00, *p* = 0.029) and *Lachnospiraceae NC2004* group (OR = 0.96, 95% CI, 0.94–0.99, *p* = 0.018) were negatively correlated with SUA levels. By contrast, *Escherichia Shigella* (OR = 1.04, 95% CI, 1.00–1.08, *p* = 0.035) showed a positive correlation with SUA levels. MR Egger’s methods provided estimates suggesting a negative association between the genetically predicted *Lachnospiraceae FCS020* group (OR = 0.93, 95% CI, 0.65–0.97, *p* = 0.027) and SUA levels. Similarly, the WM methods also suggested a negative association between the genetically predicted *Lachnospiraceae FCS020* group (OR = 0.95, 95% CI, 0.92–0.99, *p* = 0.010) and SUA levels (Figure 2). Elaborate results can be accessed in Table S7. Another visualization of the results can be found in Figure S1.

#### 3.2.2. Causal Effects of GM on Gout

**Figure 3:**
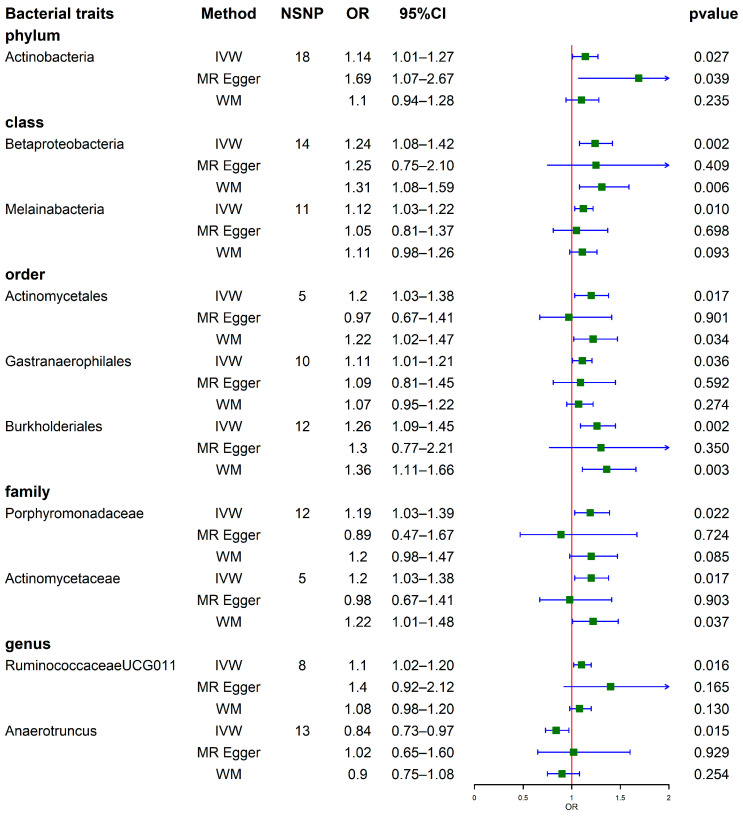
Forest plots show causal-effect estimates of GM with gout (locus-wide significance, *p* < 1 × 10^−5^).

Then, MR analysis was performed for GM and gout; Figure 3 illustrates the causal effects between 10 GM taxa and gout at 5 levels, including 1 phylum, 2 classes, 3 orders, 2 families, and 2 genera. Among the MR results at the phylum and class levels, IVW analyses revealed positive correlations between gout and *Actinobacteria* (OR = 1.14, 95% CI, 1.01–1.27, *p* = 0.027), *Betaproteobacteria* (OR = 1.24, 95% CI, 1.08–1.42, *p* = 0.002), and *Melainabacteria* (OR = 1.12, 95% CI, 1.03–1.22, *p* = 0.010). Regarding the order and family levels, IVW analyses identified 5 taxa as risk factors for gout, including *Actinomycetales* (OR = 1.20, 95% CI, 1.03–1.38, *p* = 0.017), *Gastranaerophilales* (OR = 1.11, 95% CI, 1.01–1.21, *p* = 0.036), *Burkholderiales* (OR = 1.26, 95% CI, 1.09–1.45, *p* = 0.002), *Porphyromonadaceae* (OR = 1.19, 95% CI, 1.03–1.39, *p* = 0.022), and *Actinomycetaceae* (OR = 1.20, 95% CI, 1.03–1.38, *p* = 0.017). Among these, *Actinomycetales*, *Burkholderiales* and *Actinomycetaceae* also showed significance in the results using the WM method. At the genus level, IVW analyses revealed a positive association between *RuminococcaceaeUCG011* (OR = 1.10, 95% CI, 1.02–1.20, *p* = 0.016) and gout. Conversely, *Anaerotruncus* (OR = 0.84, 95% CI, 0.73–0.97, *p* = 0.015) showed a negative correlation with gout (Figure 3). Detailed statistical the results for the 196 GM taxa are presented in Table S8. The visual representations of results can be found in Figure S1.

### 3.3. Sensitivity Analysis

Table S9 presents the results of the pleiotropy and heterogeneity assessments conducted separately for all 196 GM taxa. No evidence of horizontal pleiotropy was observed in the 5 GM taxa on SUA and 10 GM taxa on gout (*p* > 0.05). For instance, the *Lachnospiraceae FCS020* group had a value of (*p* = 0.122), *Melainabacteria* was (*p* = 0.640), and *Burkholderiales* was (*p* = 0.893). Similarly, no evidence of heterogeneity was observed in *Actinobacteria* (Q_*p* = 0.060) and family *XIII* (Q_*p* = 0.570) for SUA. Similar results were observed for other GM taxa (Table 1). The leave-one-out analysis revealed no potential outliers among the GM IVs, and the results are shown in Figure S2. The Bonferroni correction method was utilized to establish significance thresholds for multiple-testing correction. The adjusted significance thresholds of different taxonomic levels were as follows: phylum *p* = 5.56 × 10^−3^ (0.05/9), order *p* = 4.50 × 10^−3^ (0.05/20), genus *p* = 4.20 × 10^−4^ (0.05/119), family *p* = 1.56 × 10^−3^ (0.05/32), and class *p* = 3.13 × 10^−3^ (0.05/16). Under these conditions, we observed statistically significant results only for class *Betaproteobacteria* (*p* = 1.71 × 10^−3^) and the order *Burkholderiales* (*p* = 2.13 × 10^−3^) in relation to gout.

### 3.4. Results of MR Analysis (Genome-Wide Statistical Significance, p < 5 × 10^−8^)

#### 3.4.1. Results of MR Analysis with GM as a Whole

The MR analysis between the whole GM with SUA levels and gout did not uncover significant causal relationships (SUA: OR = 0.98, 95% CI, 0.96–1.00, *p* = 0.099; gout: OR = 0.96, 95% CI, 0.86–1.06, *p* = 0.436). The results of heterogeneity and pleiotropy analyses confirmed the validity of our findings (Table 2).

#### 3.4.2. Results of MR Analysis with 196 GM Taxa

**Figure 4:**
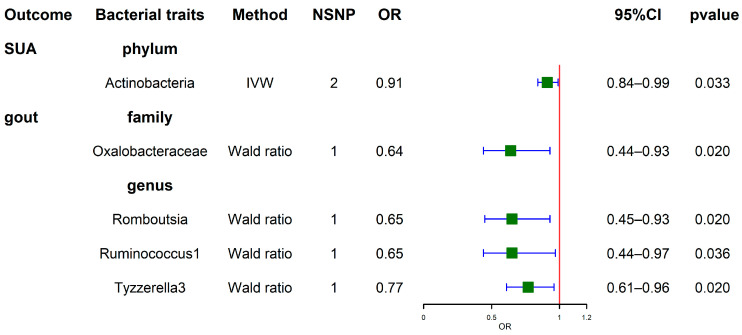
Forest plots show causal-effect estimates of GM with SUA levels and gout (genome-wide statistical significance, *p* < 5 × 10^−8^).

IVW analyses indicated that phylum *Actinobacteria* (OR = 0.91, 95% CI, 0.84–0.99, *p* = 0.033) was negatively correlated with SUA levels. This result is consistent with the findings obtained under the condition of *p* < 1 × 10^−5^. Wald ratio analyses for gout revealed negative correlations with the family *Oxalobacteraceae* (OR = 0.64, 95% CI, 0.44–0.93, *p* = 0.020), genus *Romboutsia* (OR = 0.65, 95% CI, 0.45–0.93, *p* = 0.020), genus Ruminococcus1 (OR = 0.65, 95% CI, 0.44–0.97, *p* = 0.036), and genus *Tyzzerella3* (OR = 0.77, 95% CI, 0.61–0.96, *p* = 0.020) (Figure 4). Comprehensive results are shown in Table S10.

### 3.5. Results of the Reverse MR Analysis

**Figure 5:**
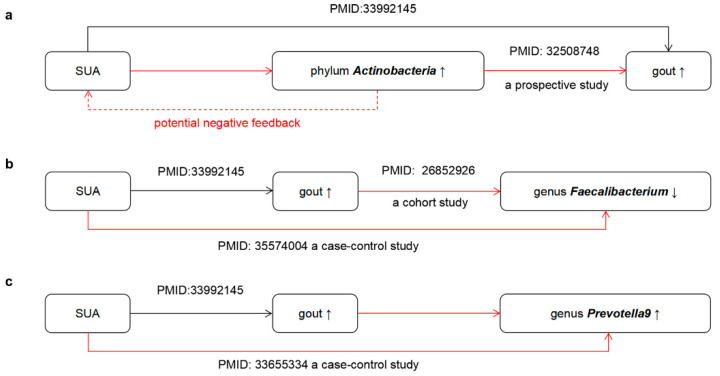
A concise overview of the primary outcomes from the MR study. The relationship between phylum Actinobacteria (**a**), genus Faecalibacterium (**b**), genus Prevotella9 (**c**) and SUA levels. Black solid arrows represent established evidence, red solid arrows represent our study’s findings, and the red dashed arrow indicates a potentially inferable conclusion. Representatives with annotated PMID numbers are supported by the literature.

All results from the reverse MR analysis are presented in Table S11. When considering GM as a whole, we did not identify any potential causality between gout or SUA levels and GM (*p* > 0.05) (Table S11). Reverse MR analysis revealed that gout affected the composition of 5 GM taxa, while SUA levels influenced the composition of 30 GM taxa (*p* < 0.05). Interestingly, Wald ratio analysis indicated that SUA levels were positively correlated with phylum *Actinobacteria* (OR = 6.48, 95% CI, 2.57–16.33, *p* = 7.42 × 10^−5^) (Table S11). Combining the existing research, we propose a potential negative feedback loop between phylum *Actinobacteria* and SUA levels (Figure 5a). The MR results also suggested that SUA levels were negative with genus *Faecalibacterium* (OR = 0.51, 95% CI, 0.31–0.83, *p* = 6.49 × 10^−3^) (Table S11, Figure 5b). Simultaneously, IVW analysis indicated a negative correlation between gout with genus *Faecalibacterium* (OR = 0.85, 95% CI, 0.74–0.99, *p* = 0.038) (Table S11, Figure 5b). Wald ratio analysis indicated that gout was positively correlated with the genus *Prevotella9* (OR = 1.78, 95% CI, 1.35–2.34, *p* = 4.13 × 10^−5^) and SUA levels were also positively correlated with the genus *Prevotella9* (OR = 2.94, 95% CI, 1.76–4.93, *p* = 4.13 × 10^−5^) (Table S11), which aligns with previous observational findings from a case–control study (Figure 5c).

## 4. Discussion

This research employed a bidirectional two-sample MR analysis to investigate the potential causal relationship between GM, gout, and SUA levels. This study provides substantial evidence indicating that genetically predicted specific GM taxa abundance plays an important role in SUA levels and the development of gout. Reverse MR analysis also suggests that gout and SUA levels influence the composition of GM. Our study provides additional supporting evidence to identify the bidirectional causal relationships between GM with gout and SUA levels.

Recently, several studies have also investigated the relationship between GM and gout. Ning Y et al. [39] reported an increased abundance of the phylum *Actinobacteria* in gout patients, which aligns with the findings of this study. This study found a novel discovery that the abundance of phylum *Actinobacteria* is negatively correlated with SUA levels. Interestingly, elevated SUA levels can also increase the abundance of phylum *Actinobacteria*, suggesting a potential negative feedback and regulatory mechanism between phylum *Actinobacteria* and SUA levels. Furthermore, the reverse MR analysis identified that SUA levels and gout simultaneously affect the abundance of the genus *Faecalibacterium* and *Prevotella9*. These findings are consistent with previous research in the field. A cohort study conducted previously found that gout patients had lower levels of the genus *Faecalibacterium* compared to healthy individuals [40]. Additionally, a case–control study in children showed a negative association between SUA levels and *Faecalibacterium* abundance [41]. SUA levels are a risk factor for the increased abundance of genus *Prevotella9* [42].

Firmicutes and Bacteroidetes are predominant in healthy adults [43]. The genus RuminococcaceaeUCG011 belongs to *Firmicutes*, and there is no direct evidence for its contribution to the development of gout in past research. In our study, we first discovered that the genus *RuminococcaceaeUCG011* is a risk factor for gout. Genus Anaerotruncus is a probiotic bacterium that also belongs to *Firmicutes* and produces butyric acid, which can provide nutrients for the human intestine, enhance intestinal immunity, promote the growth of beneficial microorganisms, and inhibit the growth of pathogenic bacteria [44]. Our findings indicate that the genus *Anaerotruncus* is a protective factor for gout. Chu et al. showed a decline in butyrate-producing bacteria in individuals with gout [45]. This result correlates with previous research, indicating that the presence of *Anaerotruncus* in the GM may have a protective effect against gout, possibly due to its probiotic properties and production of butyric acid.

The family *Porphyromonadaceae* belongs to *Bacteroides*. Our study findings are consistent with a previous cross-sectional study that also found a significant association between the family *Porphyromonadaceae* and gout [46]. This suggests that family *Porphyromonadaceae* might be a risk factor for gout. *Betaproteobacteria*, a class of the phylum *Proteobacteria*, has been identified as an opportunistic pathogen [47] and indicated a potential role as a risk factor for gout. *Melainabacteria* is a recently discovered class within the *Cyanobacteria.* Past research on the genus *Melainabacteria* has mainly focused on inflammatory diseases such as colorectal adenomas [48] and acute gastroenteritis [49]. Our study first discovered the genus *Melainabacteria* as a risk factor for gout. Furthermore, our study revealed that the presence of the genus *Lachnospiraceae FCS020* and *NC2004* act as protective factors for SUA levels. These findings highlight the potential roles of specific GM groups in SUA levels and the development of gout, further emphasizing the need for additional research to better understand underlying mechanisms and explore potential therapeutic targets.

This study possesses several strengths. MR analysis was employed to establish the causal relationship between GM with gout and SUA, thereby eliminating the influence of confounding variables and the potential for reverse causation, enhancing the ability to infer causality; genetic variation in GM was obtained from the largest available GWAS meta-analysis; palindrome SNPs were excluded during the selection of IVs to maintain the validity of the SNPs; and we employed the *F*-statistic to ensure the strength of SNPs. In the analysis, the horizontal pleiotropic and heterogenetic SNPs were detected and excluded using the MR-Egger regression test and Cochran’s Q test; MR analysis was performed using a variety of methods.

Nonetheless, this study has several limitations that need to be acknowledged. It is important to note that a significant gender disparity in gout was prevalent, with men being more commonly affected than women. Due to constraints in the available GWAS data, a subgroup analysis focusing on gender-specific effects could not be performed; the lowest taxonomic classification level as a genus in this study limited the ability to establish a more comprehensive investigation into the causal relationship between GM and gout. The GWAS data used in this study encompass populations from different ethnic backgrounds; however, the majority of the data were derived from individuals of European ancestry. Consequently, caution should be exercised when generalizing the outcomes of different ethnic backgrounds.

## 5. Conclusions

We comprehensively assessed the causal relationships between GM with gout and SUA levels. The MR analysis identified 5 bacterial taxa associated with SUA levels and 10 taxa associated with gout. The reverse MR analysis revealed that gout affects the composition of 5 GM taxa, while SUA levels influence the composition of 30 GM taxa. Notably, we propose a potential negative feedback loop between phylum *Actinobacteria* and SUA levels. Additionally, our findings indicate that SUA levels and gout simultaneously impact the abundance of the genus *Faecalibacterium* and genus *Prevotella9*. The statistical causality established by MR only establishes statistical causality, providing a degree of causal evidence for the relationship between exposure and outcome, but cannot definitively prove exact causal connections. The statistical causality established by MR cannot fully prove the exact causal associations between exposure and outcome. To establish precise causal relationships, more comprehensive research is required. Exploring the biological mechanisms underlying the mutual interactions between GM and gout or SUA levels still requires further animal experiments and population studies. Overall, our study provides further supportive evidence and valuable insights into the causal relationship between GM and the development of gout, as well as SUA levels, offering clues for its mechanistic exploration and the search for potential therapeutic targets.

## Figures and Tables

**Table 1:** Sensitivity analysis between GM with SUA and gout (locus-wide significance, *p* < 1 × 10^−5^).

Outcome	Taxonomies	GM	Q	Q_*p*	Intercept	*p*
SUA						
	phylum					
		*Actinobacteria*	25.78	0.060	−0.002	0.755
	family					
		Family *XIII*	8.62	0.570	0.007	0.161
	genus					
		*Escherichia Shigella*	15.89	0.100	0.001	0.847
		*Lachnospiraceae FCS020* group	14.11	0.370	0.004	0.122
		*Lachnospiraceae NC2004* group	14.52	0.070	−0.001	0.929
gout						
	phylum					
		*Actinobacteria*	9.92	0.871	−0.025	0.098
	class					
		*Betaproteobacteria*	12.42	0.412	−0.001	0.967
		*Melainabacteria*	9.62	0.382	0.007	0.640
	order					
		*Actinomycetales*	2.89	0.409	0.022	0.326
		*Gastranaerophilales*	10.00	0.265	0.002	0.895
		*Burkholderiales*	11.25	0.338	−0.002	0.893
	family					
		*Porphyromonadaceae*	6.76	0.748	0.017	0.372
		*Actinomycetaceae*	2.89	0.409	0.022	0.327
	genus					
		*RuminococcaceaeUCG011*	2.21	0.899	−0.032	0.300
		*Anaerotruncus*	12.52	0.326	−0.014	0.377

**Table 2:** Results of MR analysis with GM as a whole (genome-wide statistical significance, *p* < 5 × 10^−8^).

GM	Outcome	Method	NSNP	OR	95% CI	*p*	Q	Q_*p*	Intercept	*p*
Total	SUA	IVM	12	0.98	0.96–1.00	0.099	11.18	0.428		
Total	SUA	MR Egger	12	1.01	0.94–1.08	0.873	10.69	0.382	−0.003	0.513
Total	SUA	WM	12	0.99	0.96–1.02	0.430				
Total	gout	IVM	12	0.96	0.86–1.06	0.436	18.60	0.069		
Total	gout	MR Egger	12	1.00	0.70–1.44	0.997	18.49	0.047	−0.005	0.816
Total	gout	WM	12	1.02	0.91–1.14	0.787				

## Data Availability

MiBioGen repository, https://mibiogen.gcc.rug.nl/ (accessed on 15 March 2023). CKDGEN repository, http://ckdgen.imbi.uni-freiburg.de/ (accessed on 7 February 2023).

## Supplementary Materials

The following supporting information can be downloaded at: https://www.mdpi.com/article/10.3390/nu15194260/s1, File S1: Tables S1–S11; Table S1: STROBE-MR checklist of the present study; Table S2: Characteristics of data in this study; Tables S3 and S4: IVs related to GM with SUA and gout, respectively (*p* < 1 × 10^−5^); Table S5: IVs related to GM with gout and SUA (*p* < 5 × 10^−8^); Table S6: IVs of the Reverse MR analysis (*p* < 5 × 10^−8^); Tables S7 and S8: Casual effects of MR analysis between GM with SUA and gout (*p* < 1 × 10^−5^); Table S9: Results of sensitivity analysis (*p* < 1 × 10^−5^); Table S10: Casual effects of MR analysis between GM with SUA and gout (*p* < 5 × 10^−8^); Table S11: Reverse MR analysis Results (*p* < 5 × 10^−8^); Figure S1: Visualization of results, including forest plots, scatter plots and funnel plots; Figure S2: Visualization of Leave-One-Out analysis results in MR analysis.
