# Supplementary material for: Causal Relationship between Gut Microbiota and Gout: A Two-Sample Mendelian Randomization Study
Source: Nutrients. 2023 Oct 5;15(19):4260. doi: 10.3390/nu15194260 (PMC10574468; doi:10.3390/nu15194260)
Supplement: Supplementary file 1 [file nutrients-15-04260-s001.zip › Figure_S1.pdf]

Forest plot

SUA

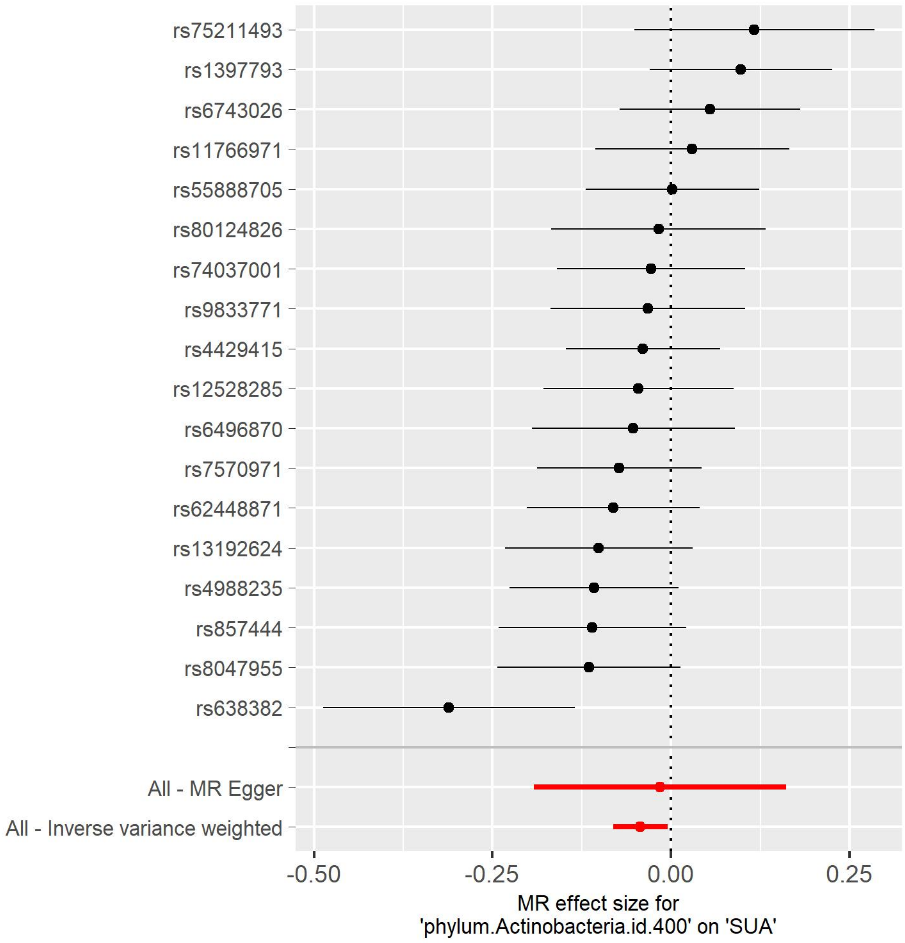

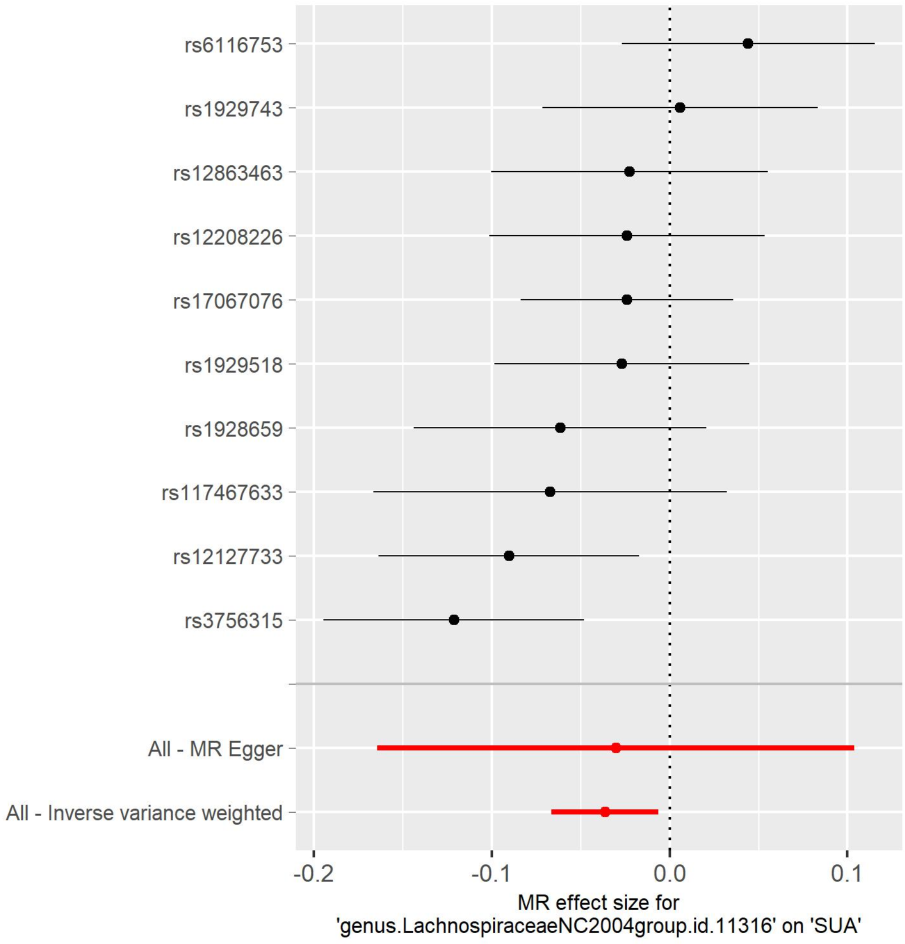

rs6116753
rs1929743
rs12863463
rs12208226
rs17067076
rs1929518
rs1928659
rs117467633
rs12127733
rs3756315
All - MR Egger
All - Inverse variance weighted
-0.2
-0.1
0.0
0.1
MR effect size for
'genus.LachnospiraceaeNC2004group.id.11316' on 'SUA'

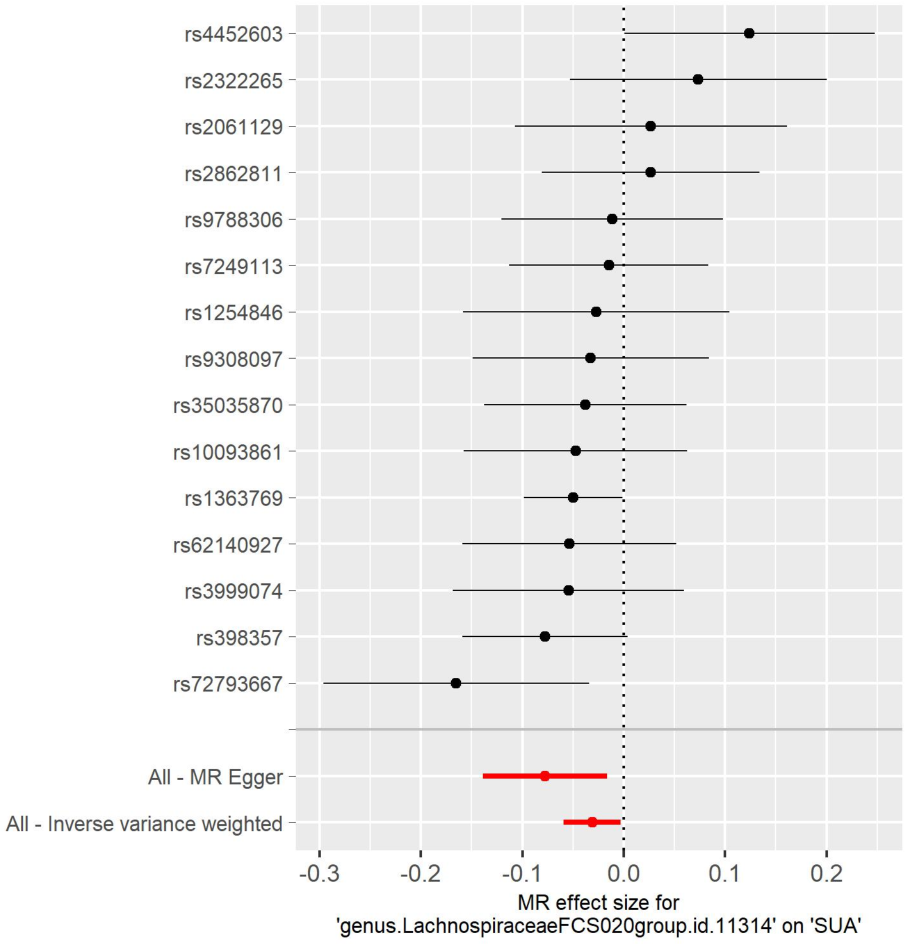
rs4452603
rs2322265
rs2061129
rs2862811
rs9788306
rs7249113
rs1254846
rs9308097
rs35035870
rs10093861
rs1363769
rs62140927
rs3999074
rs398357
rs72793667
All - MR Egger
All - Inverse variance weighted
-0.3
-0.2
-0.1
0.0
0.1
0.2
MR effect size for
'genus.LachnospiraceaeFCS020group.id.11314' on 'SUA'

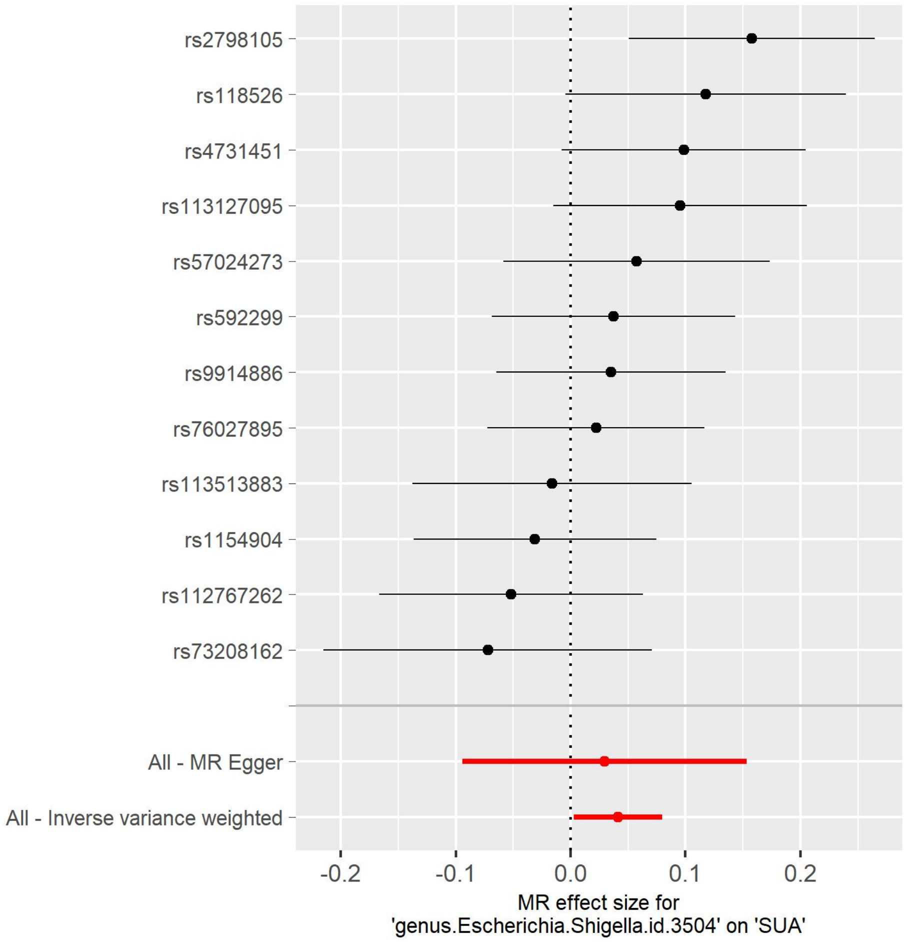

rs2798105
rs118526
rs4731451
rs113127095
rs57024273
rs592299
rs9914886
rs76027895
rs113513883
rs1154904
rs112767262
rs73208162
All - MR Egger
All - Inverse variance weighted
-0.2
-0.1
0.0
0.1
0.2
MR effect size for
'genus.Escherichia.Shigella.id.3504' on 'SUA'

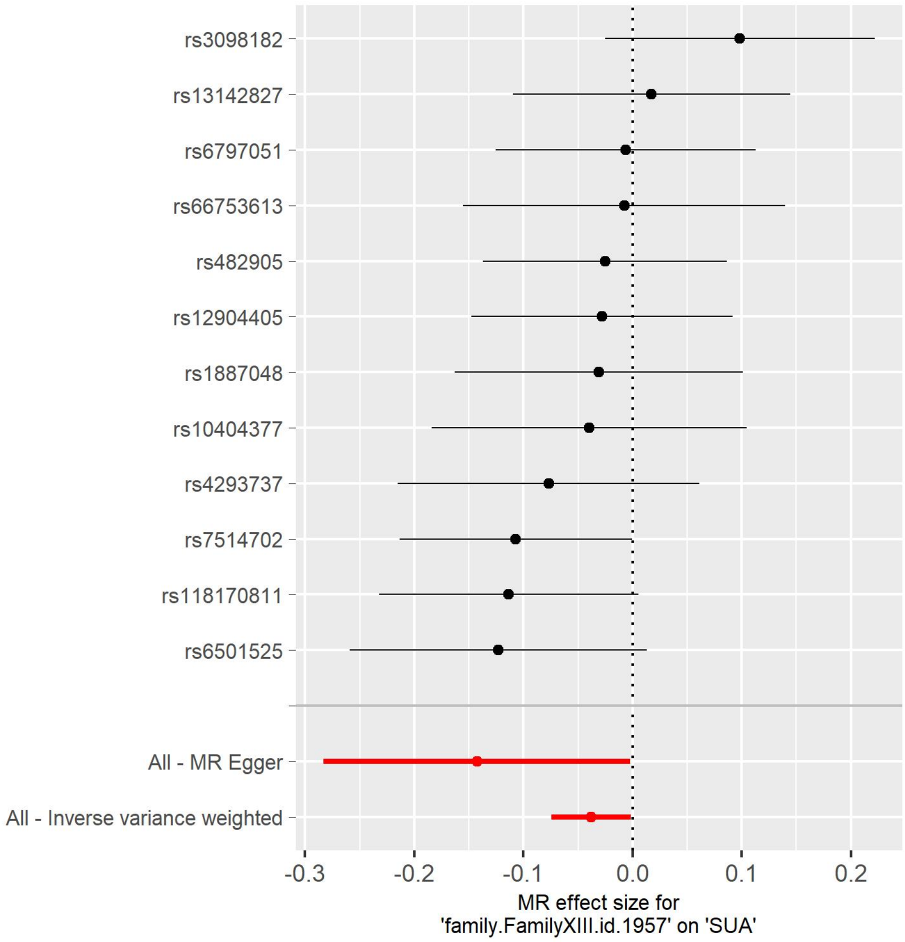
rs3098182
rs13142827
rs6797051
rs66753613
rs482905
rs12904405
rs1887048
rs10404377
rs4293737
rs7514702
rs118170811
rs6501525
All - MR Egger
All - Inverse variance weighted
-0.3
-0.2
-0.1
0.0
0.1
0.2
MR effect size for
'family.FamilyXIII.id.1957' on 'SUA'

gout

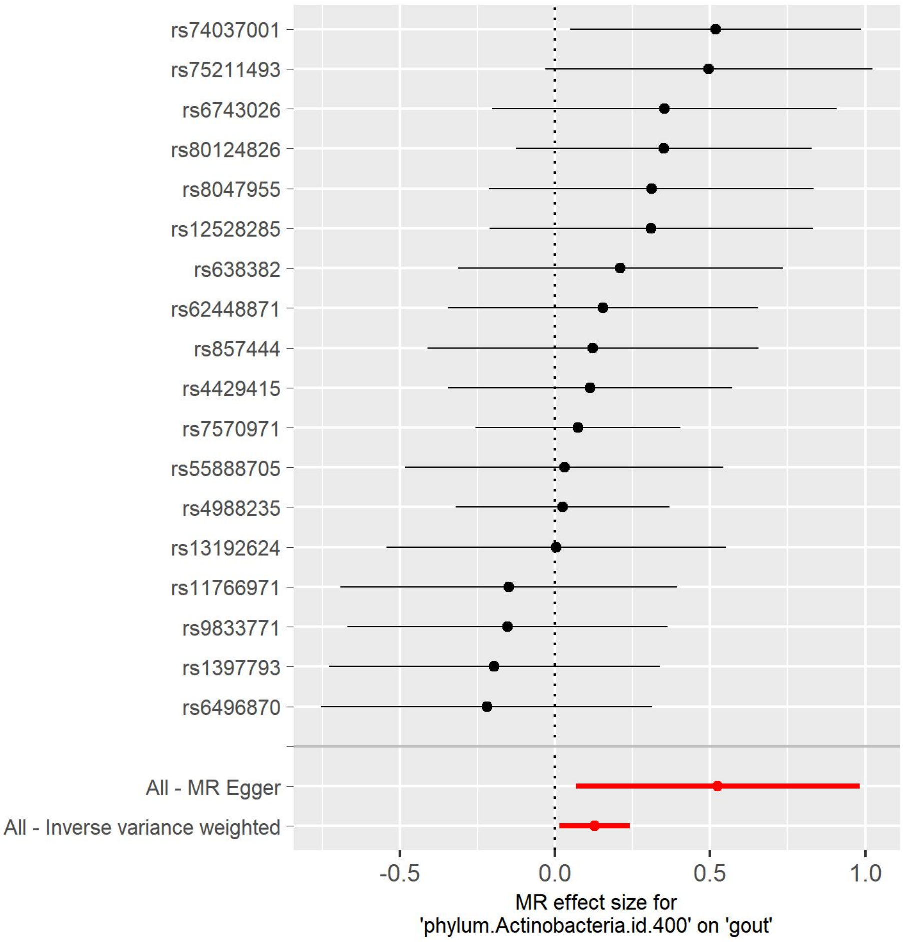
rs74037001
rs75211493
rs6743026
rs80124826
rs8047955
rs12528285
rs638382
rs62448871
rs857444
rs4429415
rs7570971
rs55888705
rs4988235
rs13192624
rs11766971
rs9833771
rs1397793
rs6496870
All - MR Egger
All - Inverse variance weighted
-0.5
0.0
0.5
1.0
MR effect size for
'phylum.Actinobacteria.id.400' on 'gout'

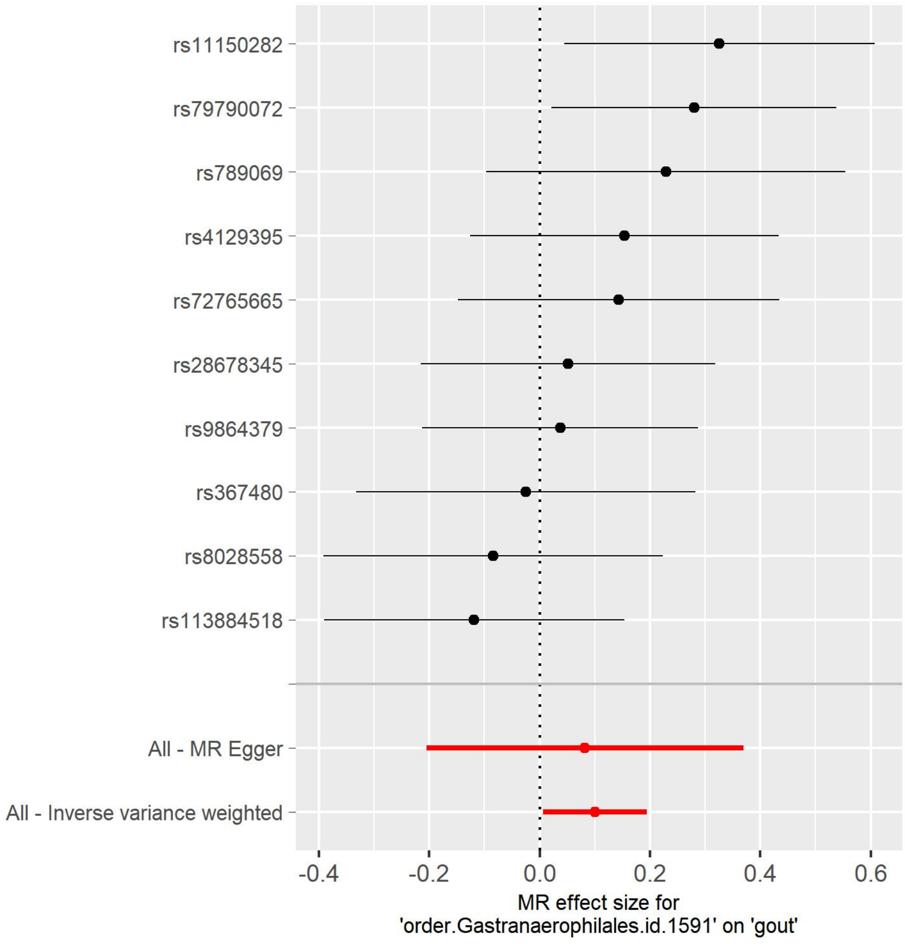
rs11150282
rs79790072
rs789069
rs4129395
rs72765665
rs28678345
rs9864379
rs367480
rs8028558
rs113884518
All - MR Egger
All - Inverse variance weighted
-0.4
-0.2
0.0
0.2
0.4
0.6
MR effect size for
'order.Gastranaerophilales.id.1591' on 'gout'

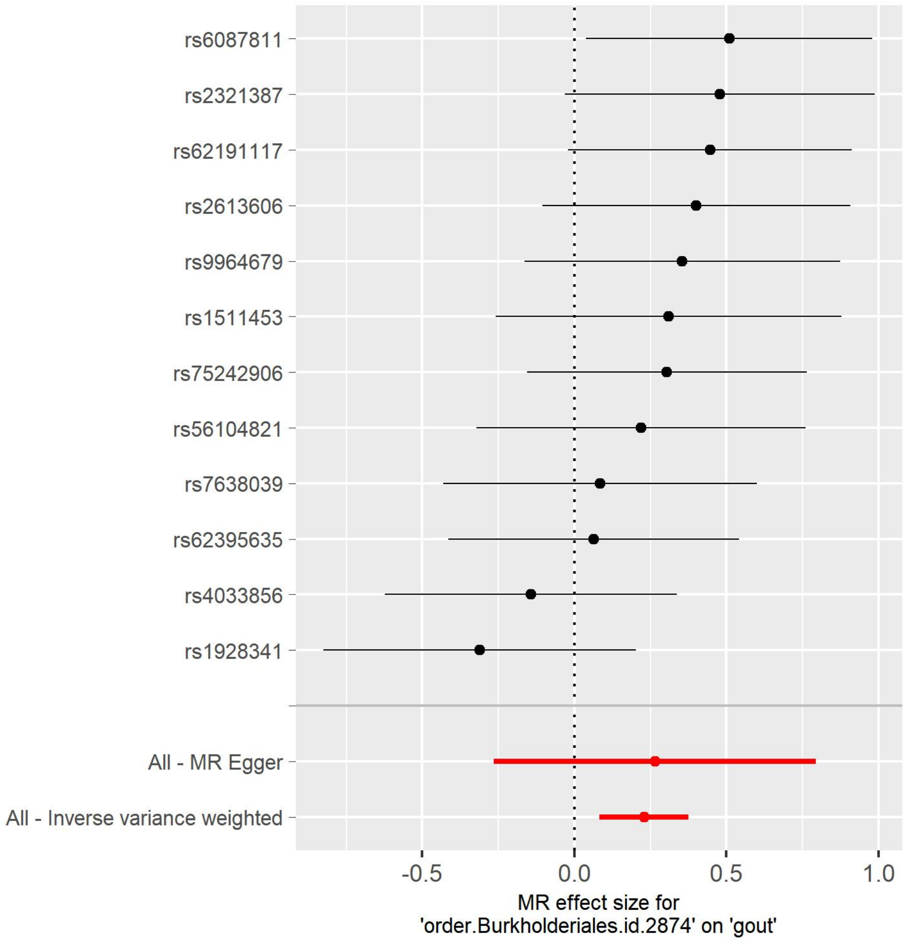
rs6087811
rs2321387
rs62191117
rs2613606
rs9964679
rs1511453
rs75242906
rs56104821
rs7638039
rs62395635
rs4033856
rs1928341
All - MR Egger
All - Inverse variance weighted
-0.5
0.0
0.5
1.0
MR effect size for
'order.Burkholderiales.id.2874' on 'gout'

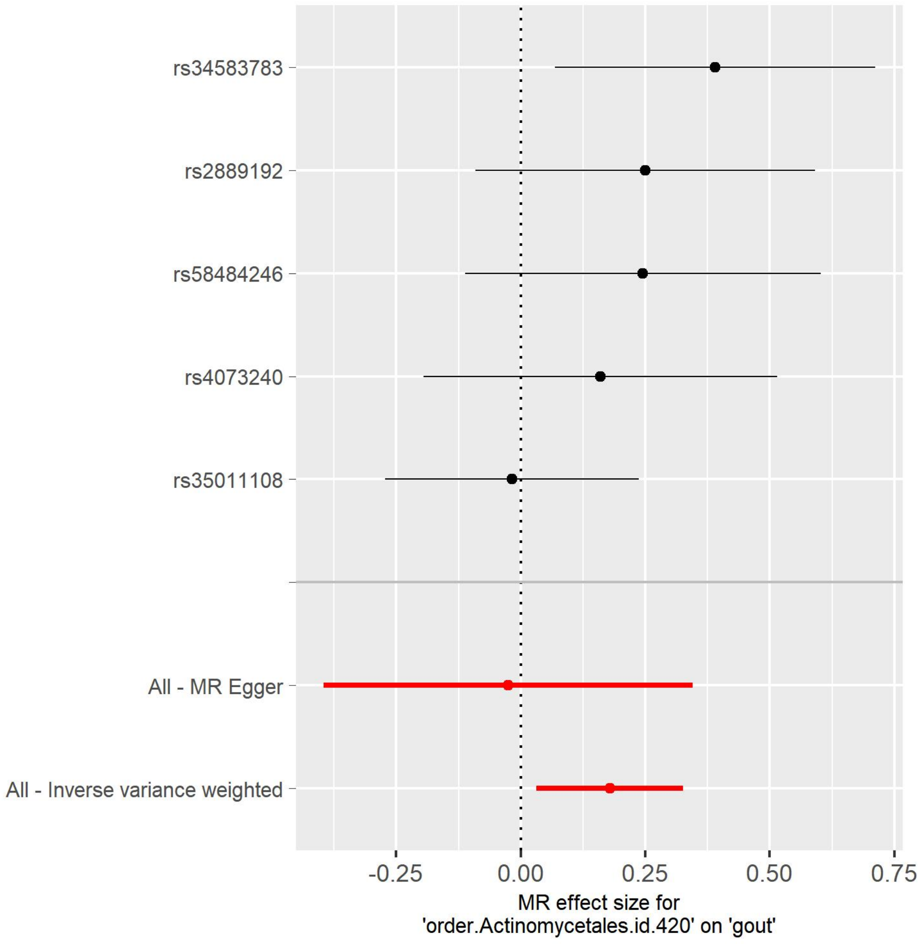
rs34583783
rs2889192
rs58484246
rs4073240
rs35011108
All - MR Egger
All - Inverse variance weighted
-0.25
0.00
0.25
0.50
0.75
MR effect size for
'order.Actinomycetales.id.420' on 'gout'

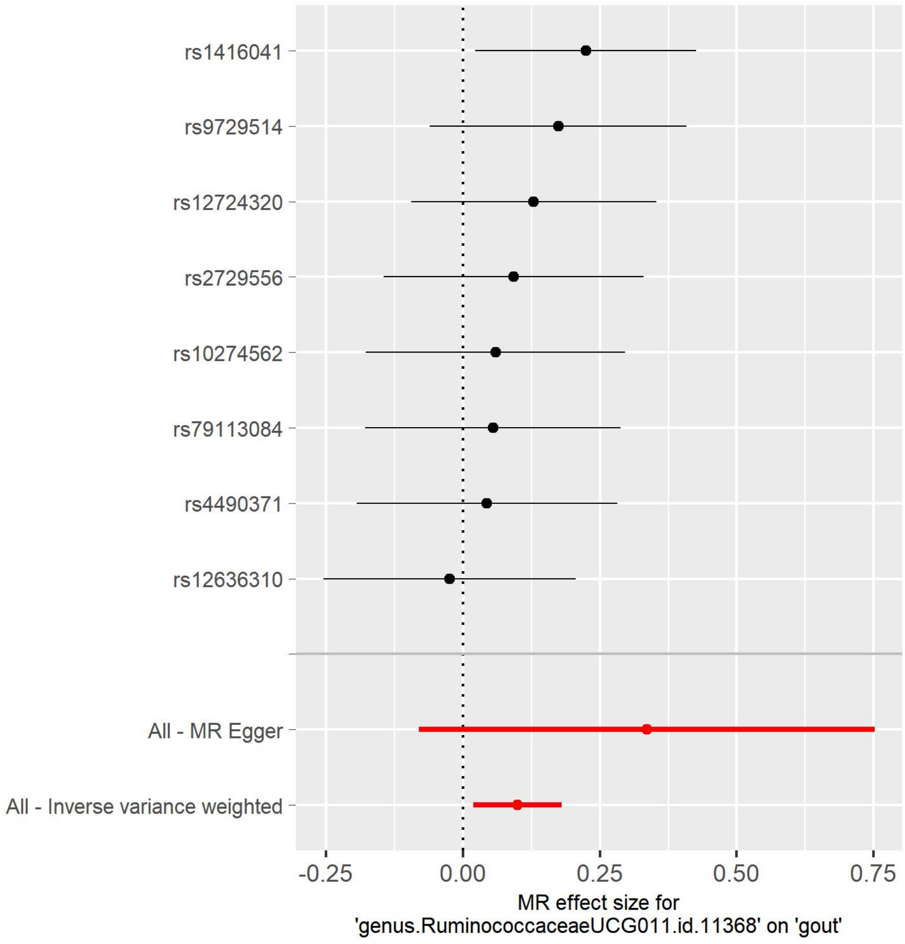

rs1416041
rs9729514
rs12724320
rs2729556
rs10274562
rs79113084
rs4490371
rs12636310
All - MR Egger
All - Inverse variance weighted
-0.25
0.00
0.25
0.50
0.75
MR effect size for
'genus.RuminococcaceaeUCG011.id.11368' on 'gout'

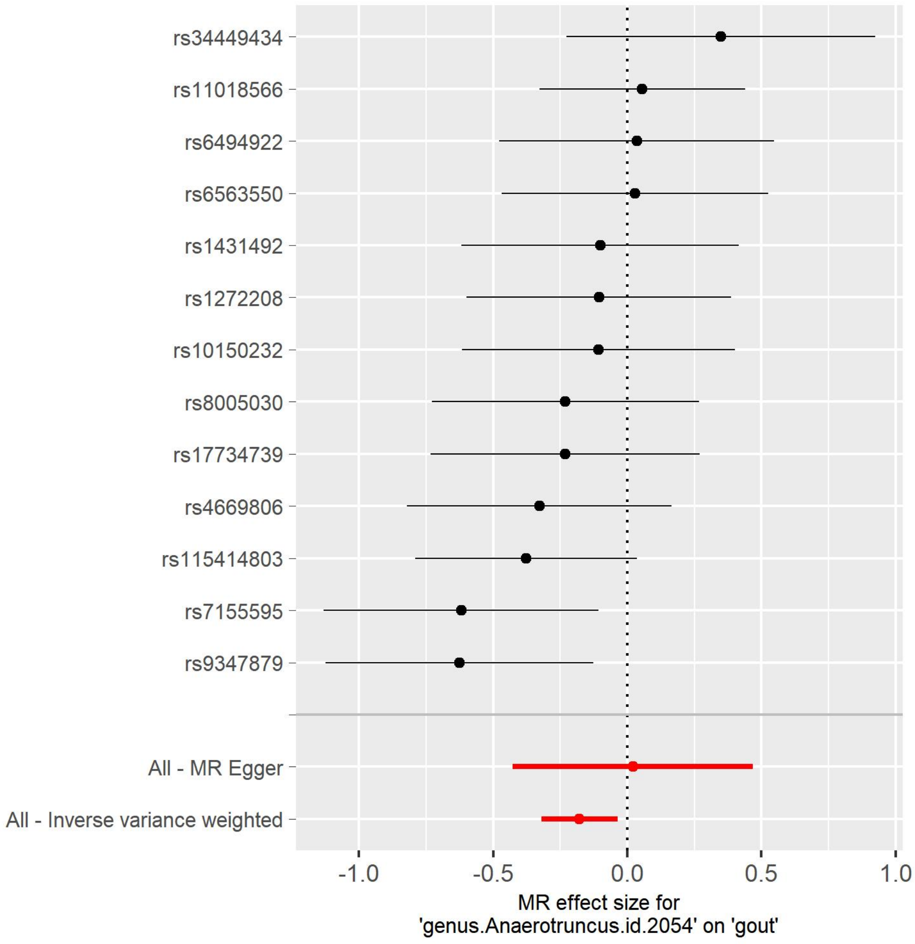
rs34449434
rs11018566
rs6494922
rs6563550
rs1431492
rs1272208
rs10150232
rs8005030
rs17734739
rs4669806
rs115414803
rs7155595
rs9347879
All - MR Egger
All - Inverse variance weighted
-1.0
-0.5
0.0
0.5
1.0
MR effect size for
'genus.Anaerotruncus.id.2054' on 'gout'

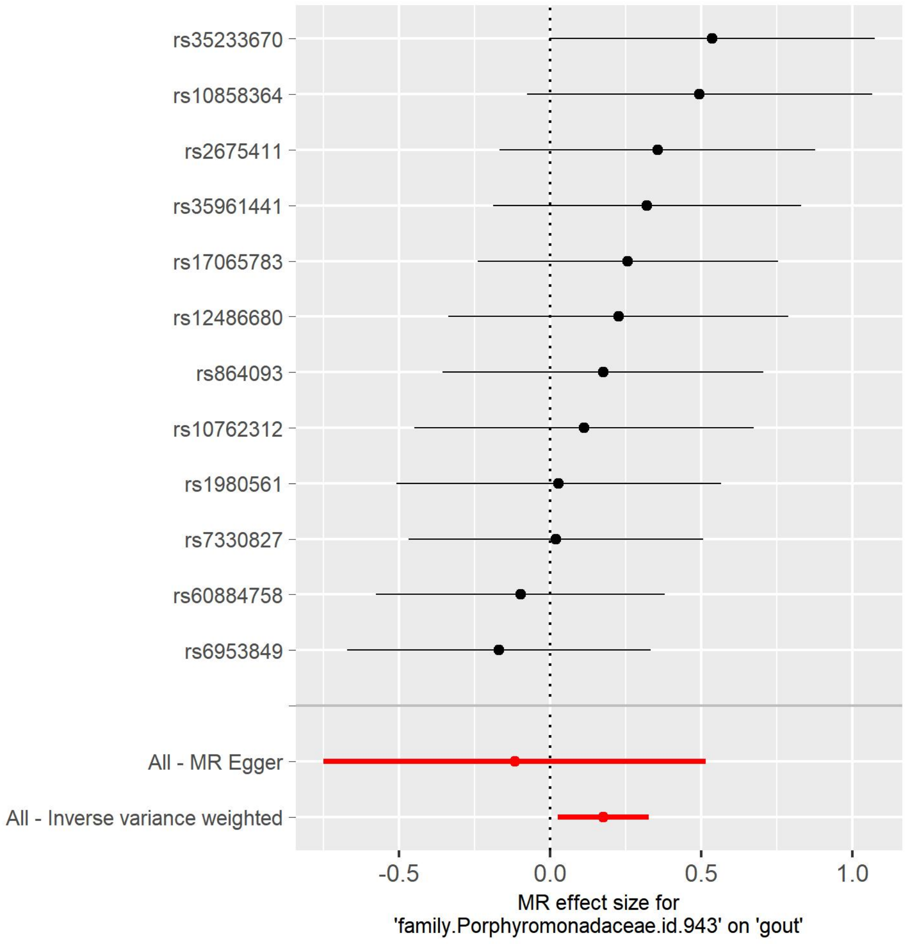
rs35233670
rs10858364
rs2675411
rs35961441
rs17065783
rs12486680
rs864093
rs10762312
rs1980561
rs7330827
rs60884758
rs6953849
All - MR Egger
All - Inverse variance weighted
-0.5
0.0
0.5
1.0
MR effect size for
'family.Porphyromonadaceae.id.943' on 'gout'

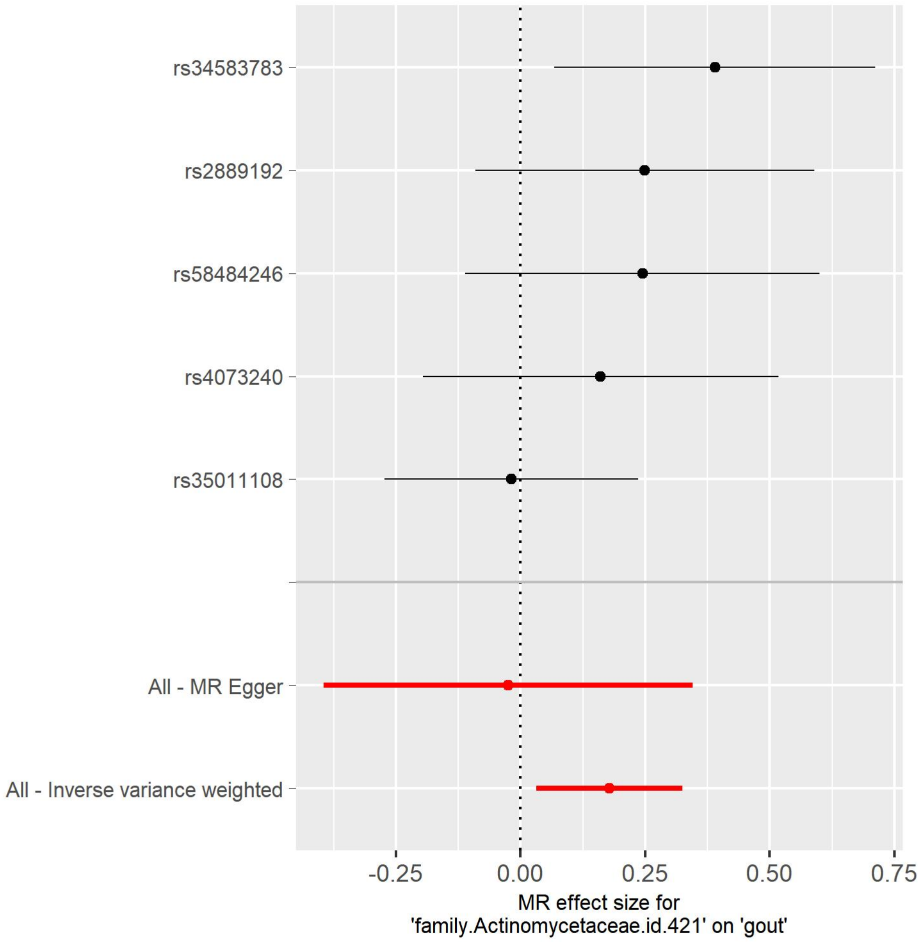

rs34583783
rs2889192
rs58484246
rs4073240
rs35011108
All - MR Egger
All - Inverse variance weighted
-0.25
0.00
0.25
0.50
0.75
MR effect size for
'family.Actinomycetaceae.id.421' on 'gout'

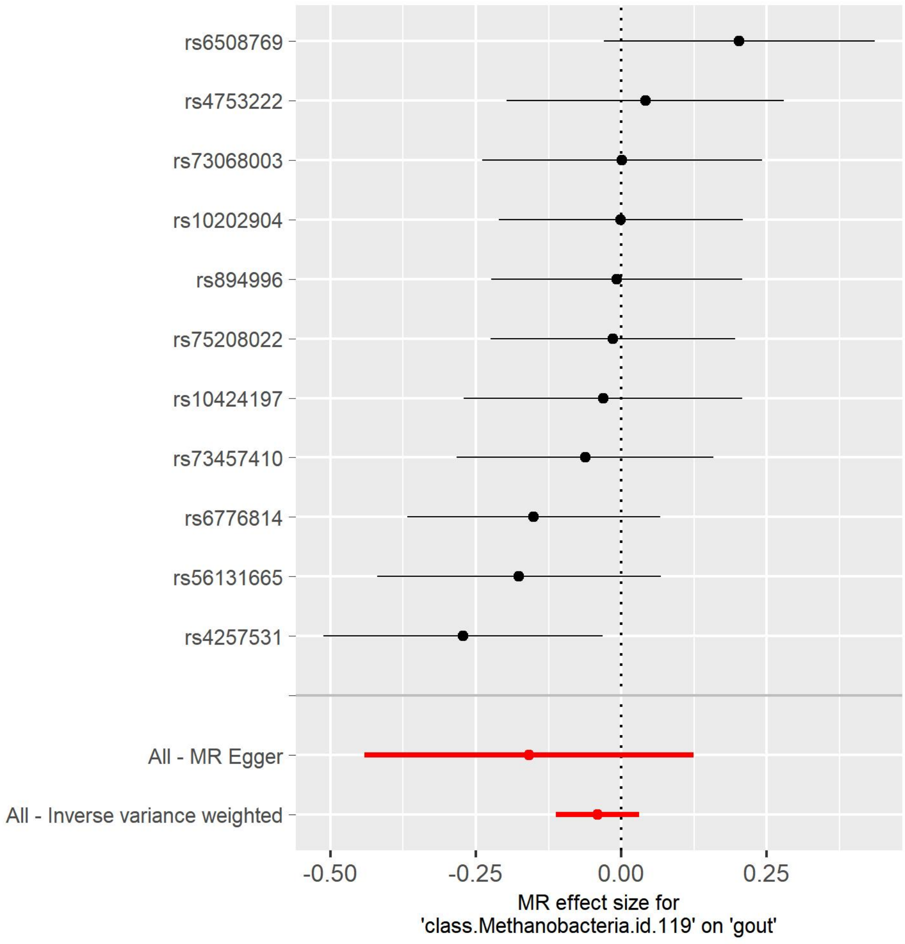
rs6508769
rs4753222
rs73068003
rs10202904
rs894996
rs75208022
rs10424197
rs73457410
rs6776814
rs56131665
rs4257531
All - MR Egger
All - Inverse variance weighted
-0.50
-0.25
0.00
0.25
MR effect size for
'class.Methanobacteria.id.119' on 'gout'

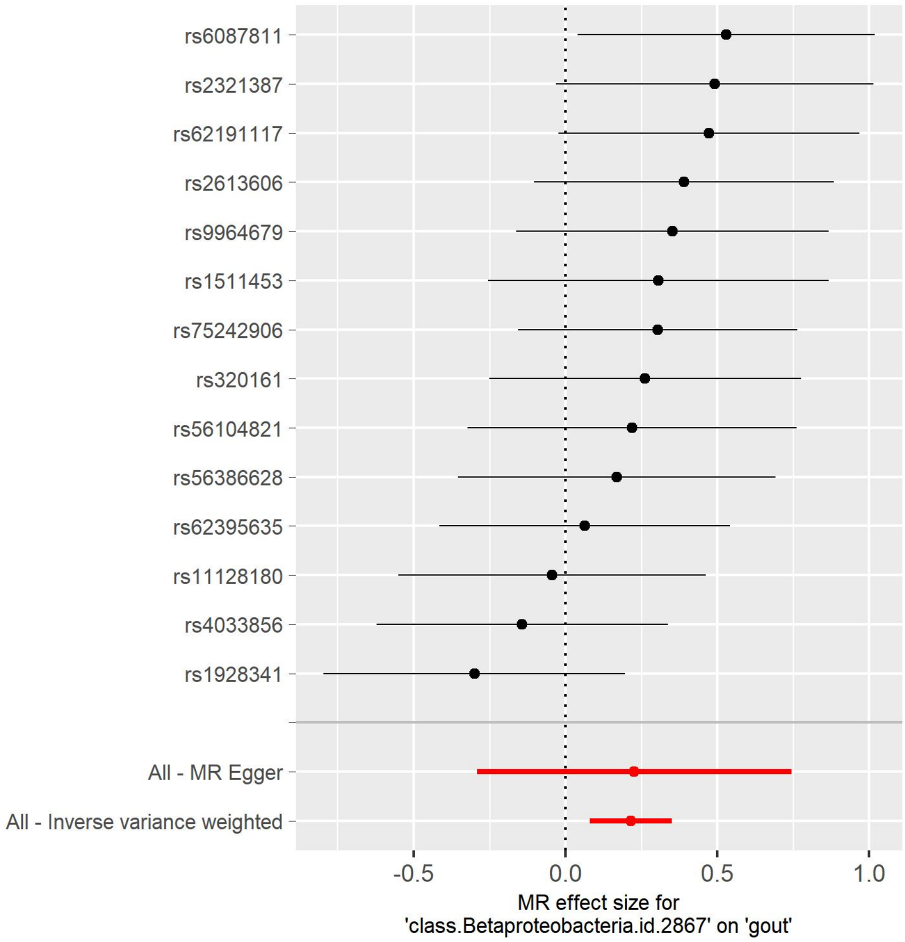

Scatter plot

SUA

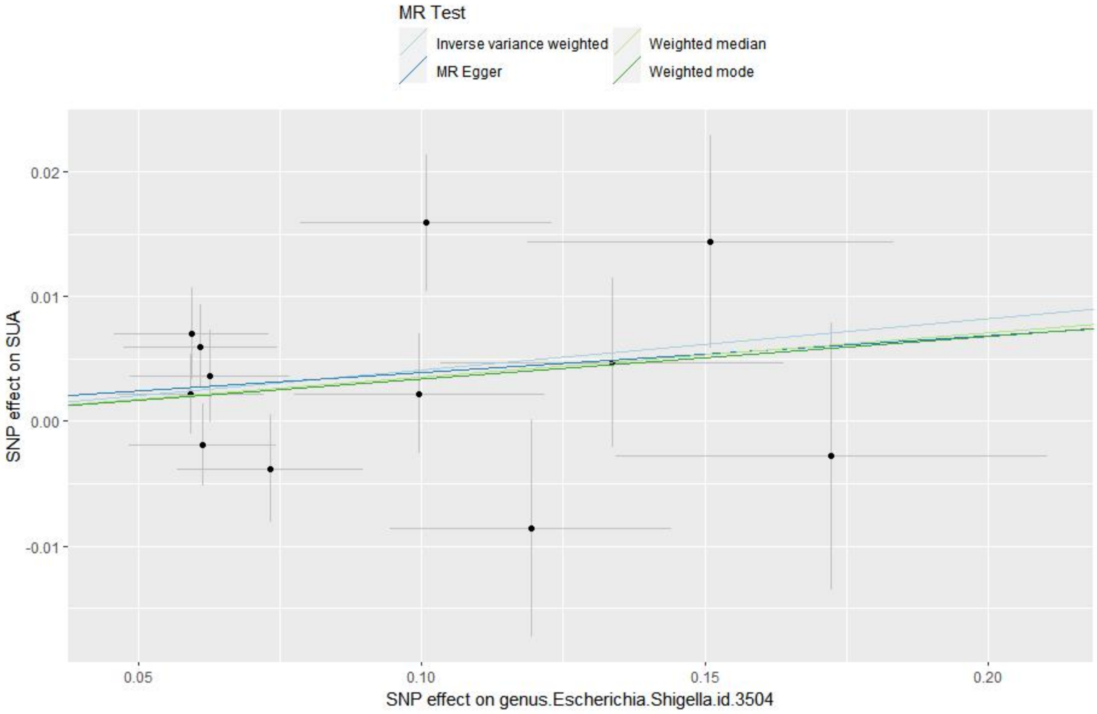
MR Test
Inverse variance weighted
Weighted median
MR Egger
Weighted mode
0.02
0.01
0.00
-0.01
SNP effect on SUA
0.05
0.10
0.15
0.20
SNP effect on genus.Escherichia.Shigella.id.3504

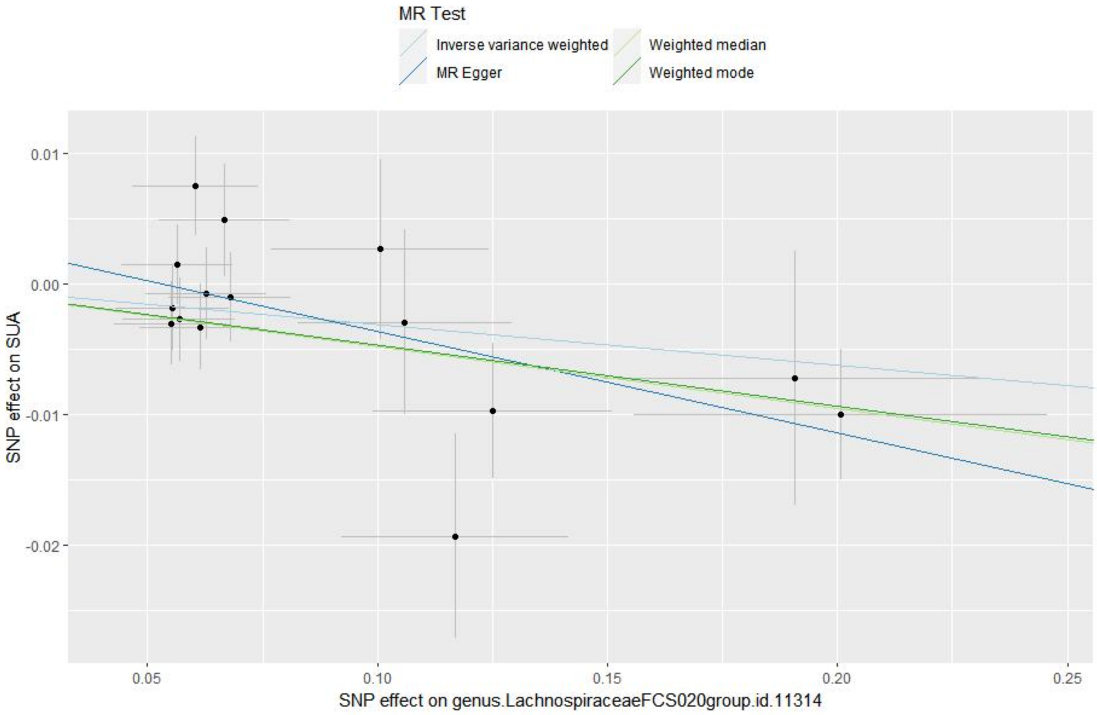
MR Test
Inverse variance weighted
Weighted median
MR Egger
Weighted mode
0.01
0.00
-0.01
-0.02
SNP effect on SUA
0.05
0.10
0.15
0.20
0.25
SNP effect on genus.LachnospiraceaeFCS020group.id.11314

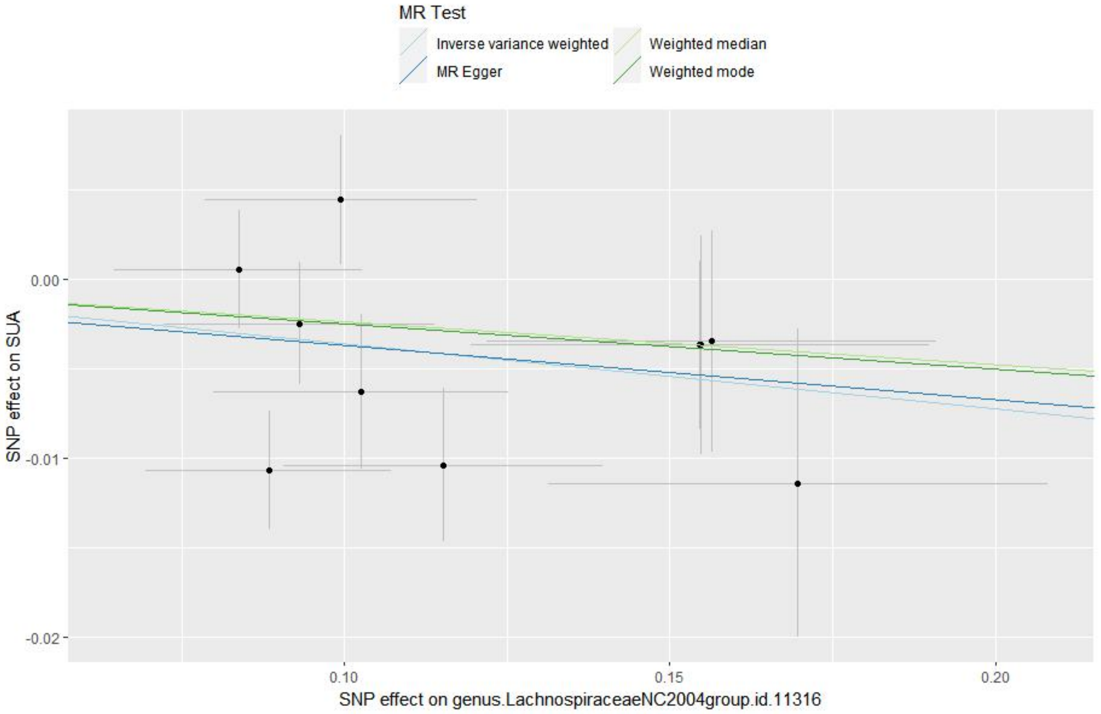
MR Test
Inverse variance weighted
MR Egger
Weighted median
Weighted mode
SNP effect on SUA
0.00
-0.01
-0.02
0.10
0.15
0.20
SNP effect on genus.LachnospiraceaeNC2004group.id.11316

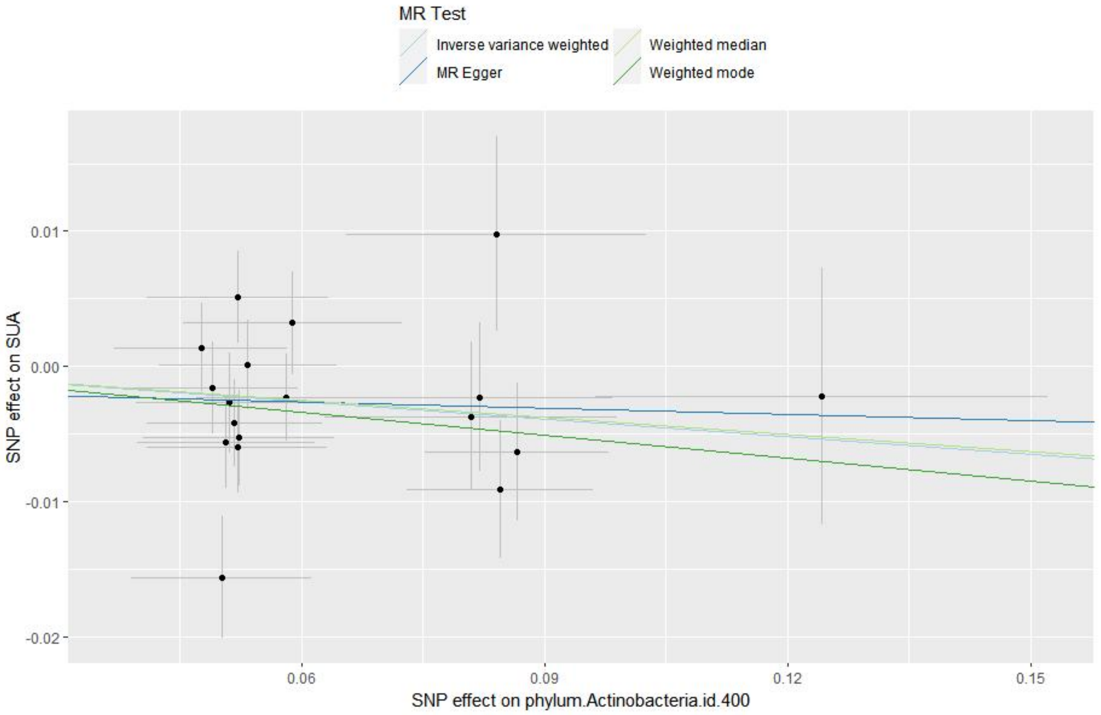
MR Test
Inverse variance weighted
MR Egger
Weighted median
Weighted mode
SNP effect on SUA
0.01
0.00
-0.01
-0.02
0.06
0.09
0.12
0.15
SNP effect on phylum.Actinobacteria.id.400

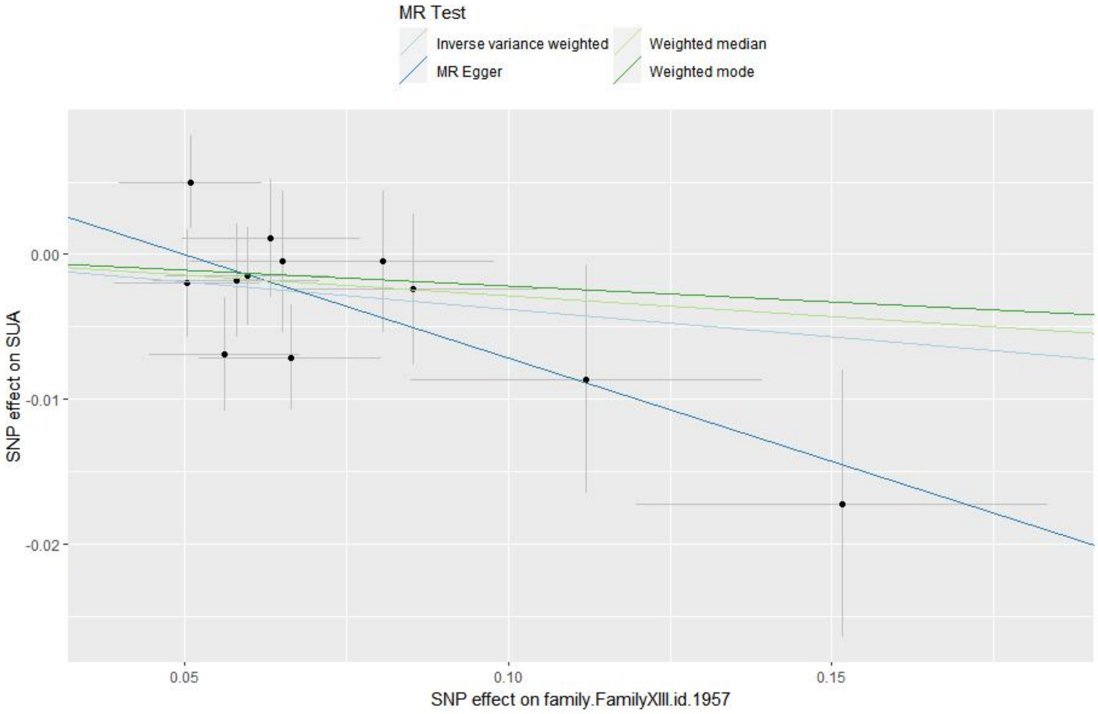
MR Test
Inverse variance weighted
MR Egger
Weighted median
Weighted mode
0.00
-0.01
-0.02
SNP effect on SUA
0.05
0.10
0.15
SNP effect on family.FamilyXIII.id.1957

Gout

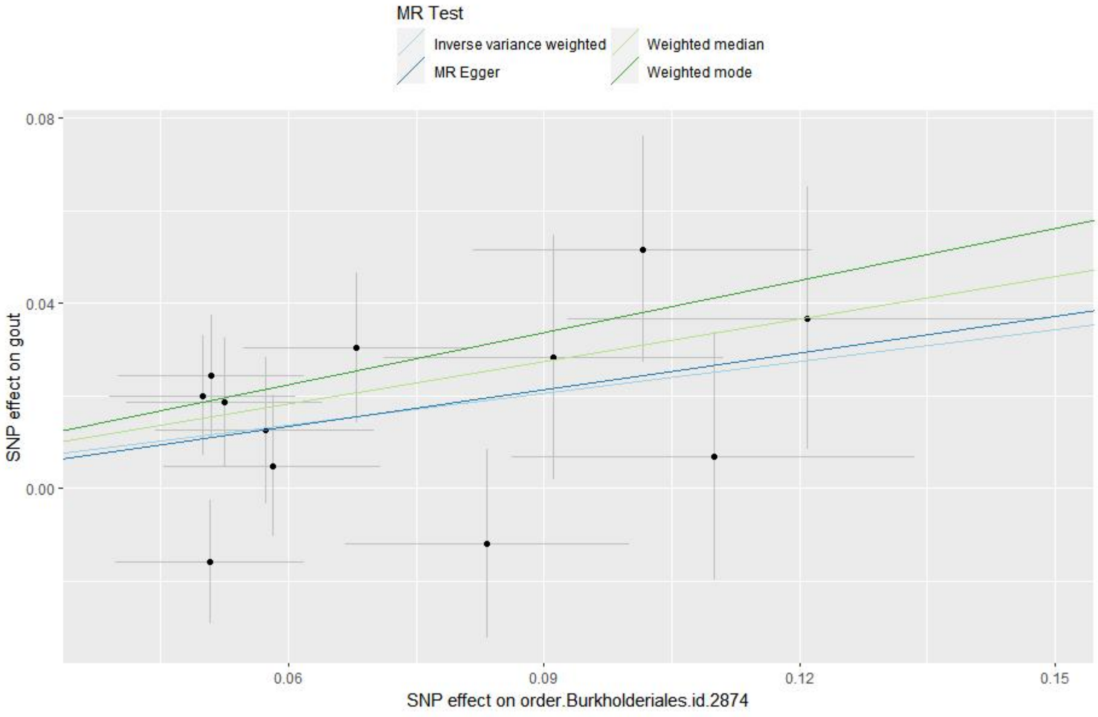
MR Test
Inverse variance weighted
MR Egger
Weighted median
Weighted mode
0.08
0.04
0.00
SNP effect on gout
0.06
0.09
0.12
0.15
SNP effect on order.Burkholderiales.id.2874

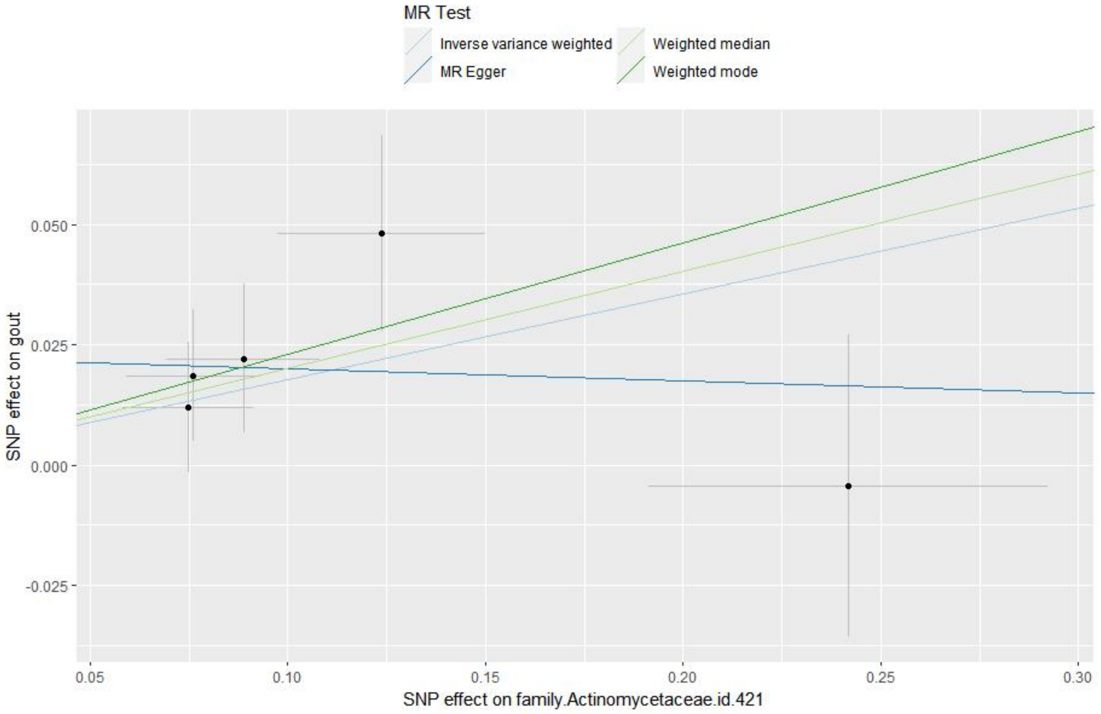
MR Test
Inverse variance weighted
MR Egger
Weighted median
Weighted mode
SNP effect on gout
0.050
0.025
0.000
-0.025
0.05
0.10
0.15
0.20
0.25
0.30
SNP effect on family.Actinomycetaceae.id.421

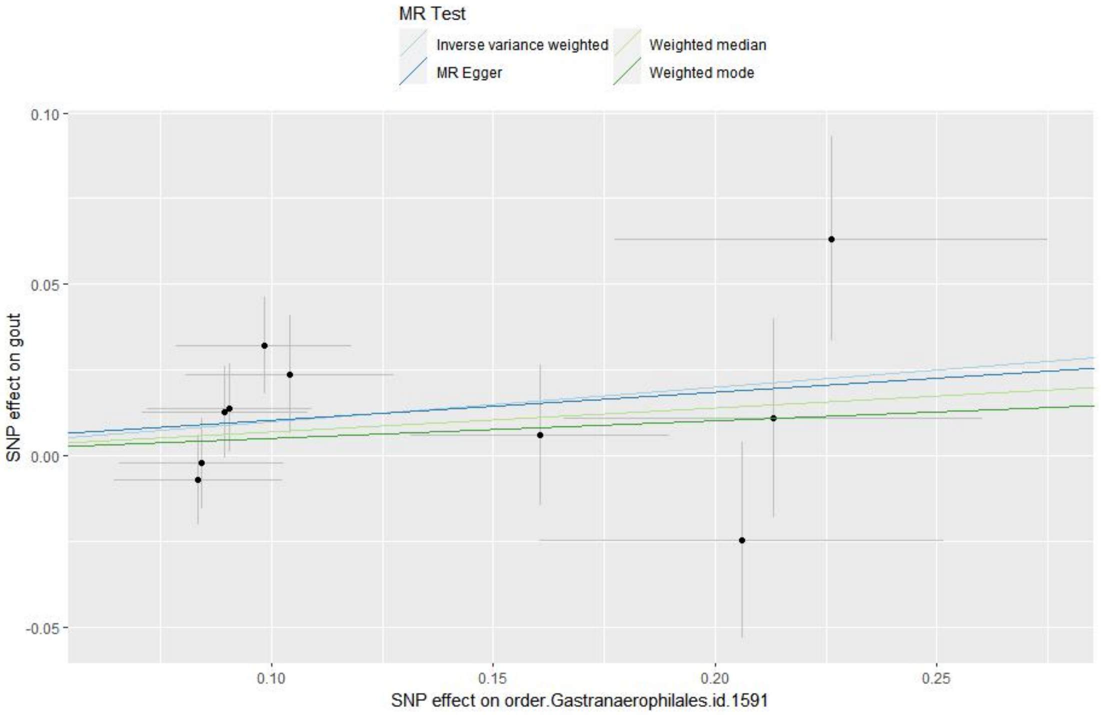
MR Test
Inverse variance weighted
MR Egger
Weighted median
Weighted mode
SNP effect on gout
0.10
0.05
0.00
-0.05
0.10
0.15
0.20
0.25
SNP effect on order.Gastranaerophilales.id.1591

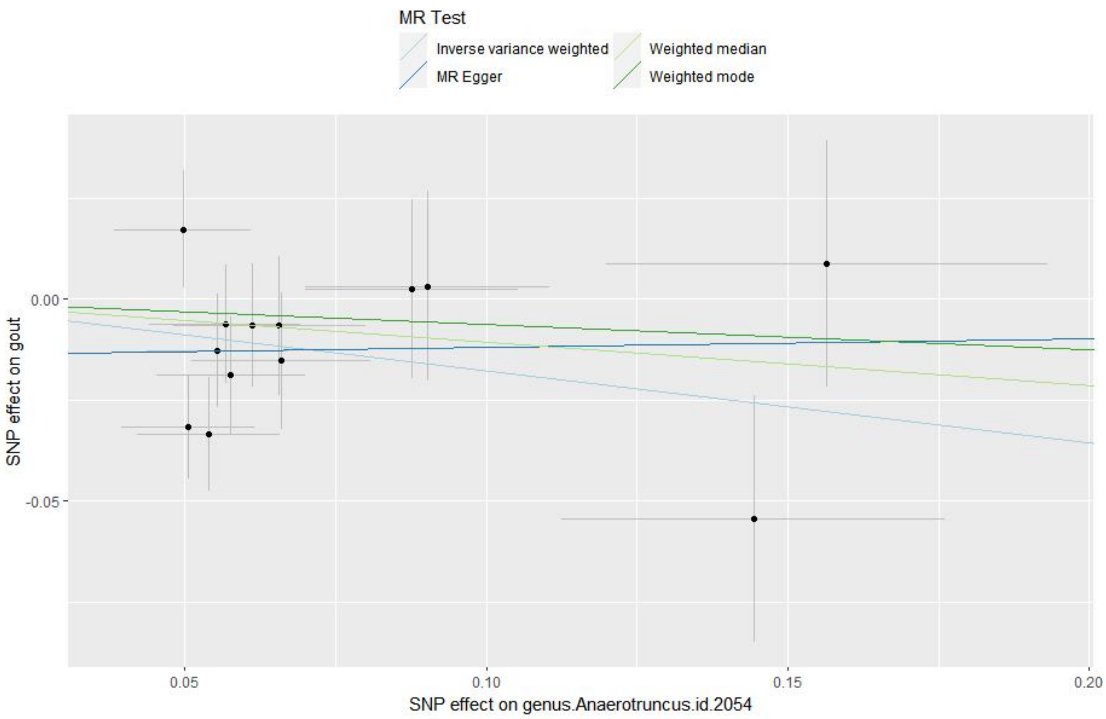
MR Test
Inverse variance weighted
MR Egger
Weighted median
Weighted mode
0.00
-0.05
SNP effect on gout
0.05
0.10
0.15
0.20
SNP effect on genus.Anaerotruncus.id.2054

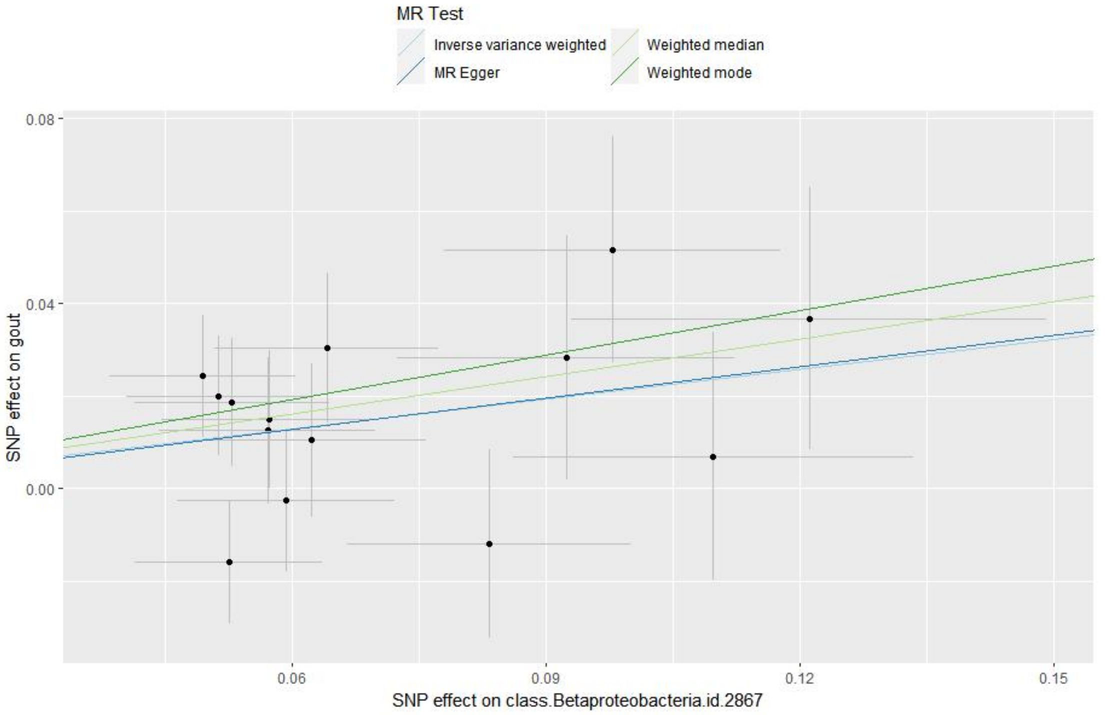
MR Test
Inverse variance weighted
MR Egger
Weighted median
Weighted mode
0.08
0.04
0.00
SNP effect on gout
0.06
0.09
0.12
0.15
SNP effect on class.Betaproteobacteria.id.2867

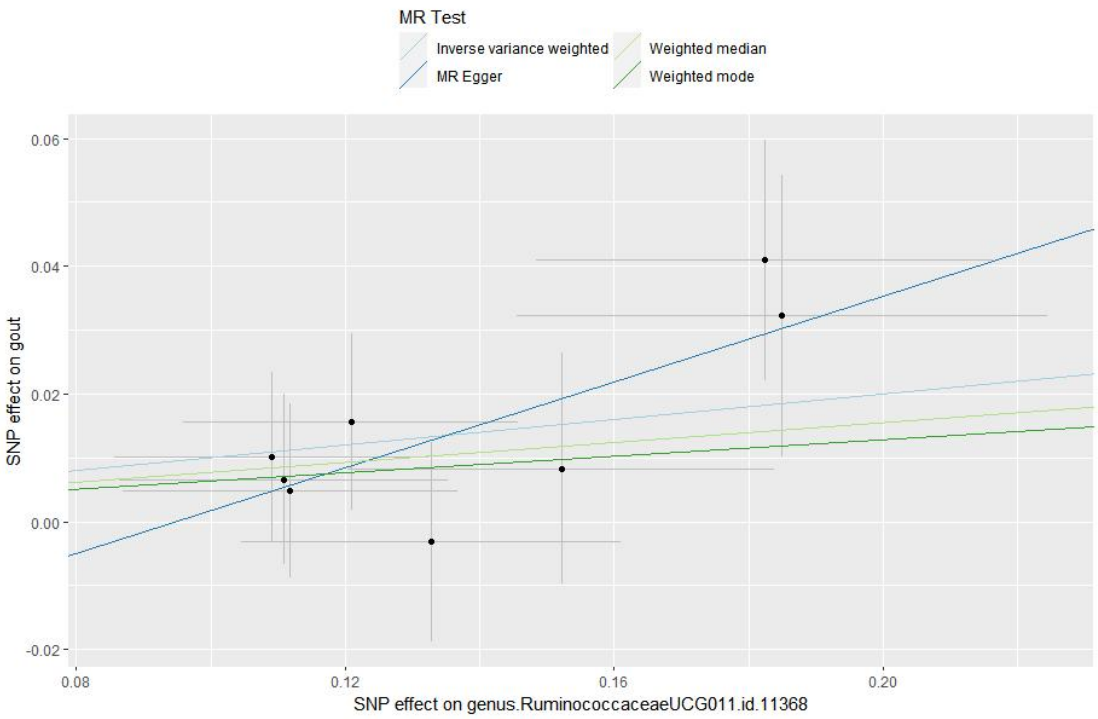
MR Test
Inverse variance weighted
MR Egger
Weighted median
Weighted mode
SNP effect on gout
0.06
0.04
0.02
0.00
-0.02
0.08
0.12
0.16
0.20
SNP effect on genus.RuminococcaceaeUCG011.id.11368

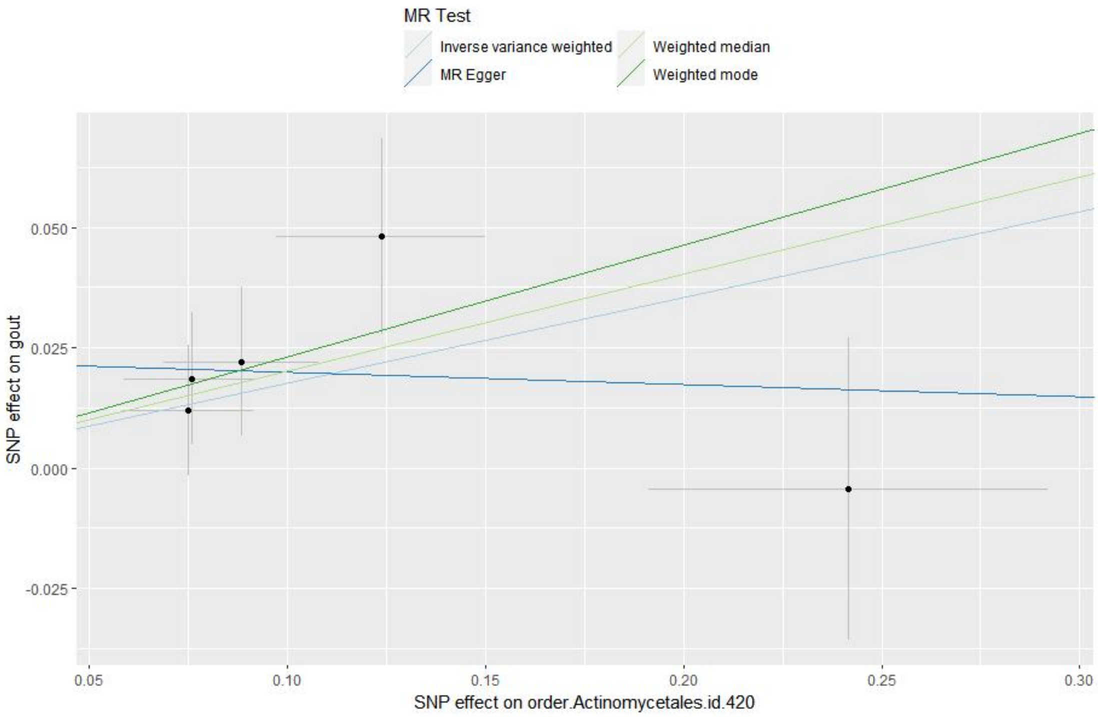
MR Test
Inverse variance weighted
MR Egger
Weighted median
Weighted mode
SNP effect on gout
0.050
0.025
0.000
-0.025
0.05
0.10
0.15
0.20
0.25
0.30
SNP effect on order.Actinomycetales.id.420

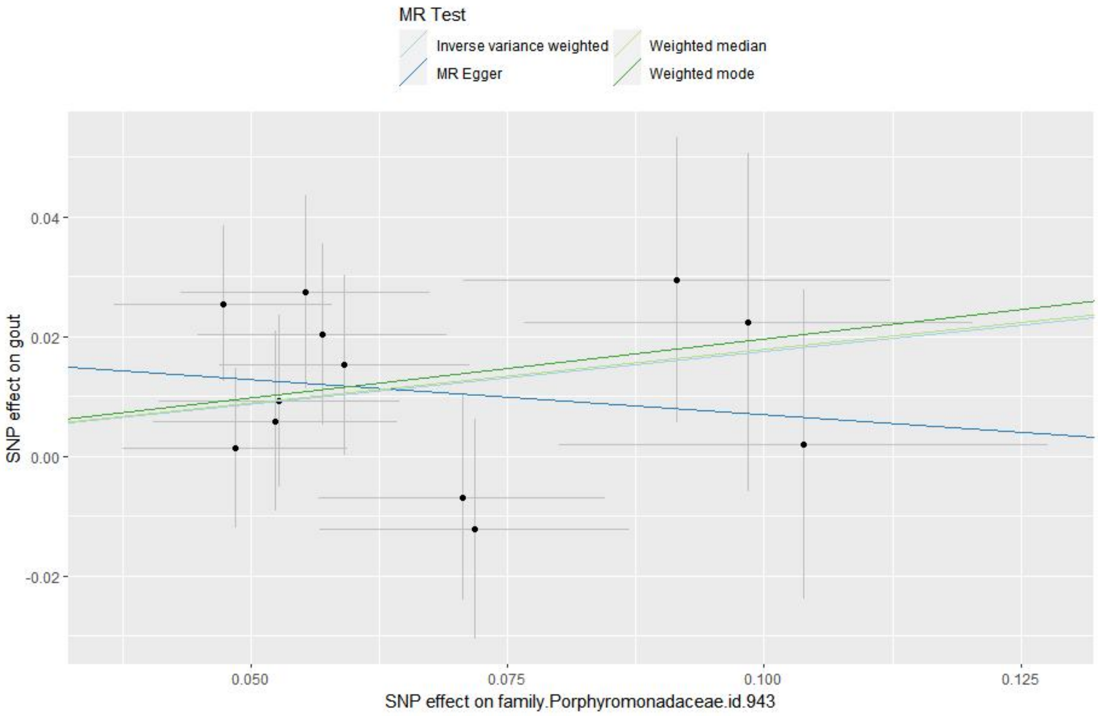
MR Test
Inverse variance weighted
Weighted median
MR Egger
Weighted mode
0.04
0.02
0.00
-0.02
SNP effect on gout
0.050
0.075
0.100
0.125
SNP effect on family.Porphyromonadaceae.id.943

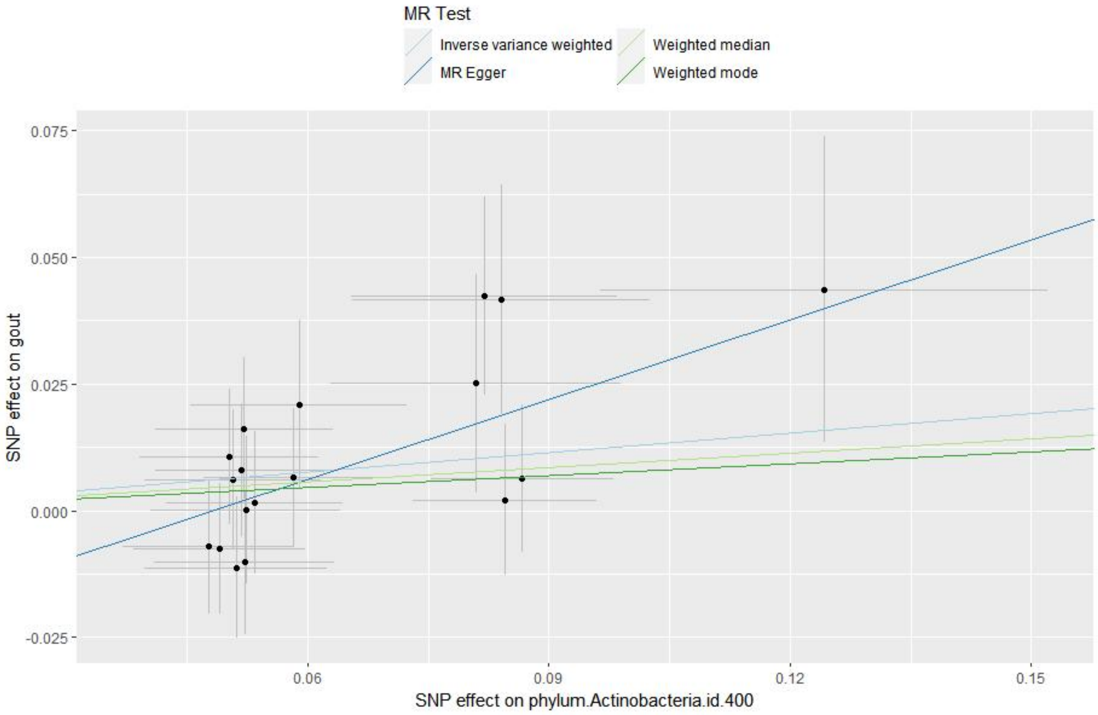
MR Test
Inverse variance weighted
Weighted median
MR Egger
Weighted mode
0.075
0.050
0.025
0.000
-0.025
SNP effect on gout
0.06
0.09
0.12
0.15
SNP effect on phylum.Actinobacteria.id.400

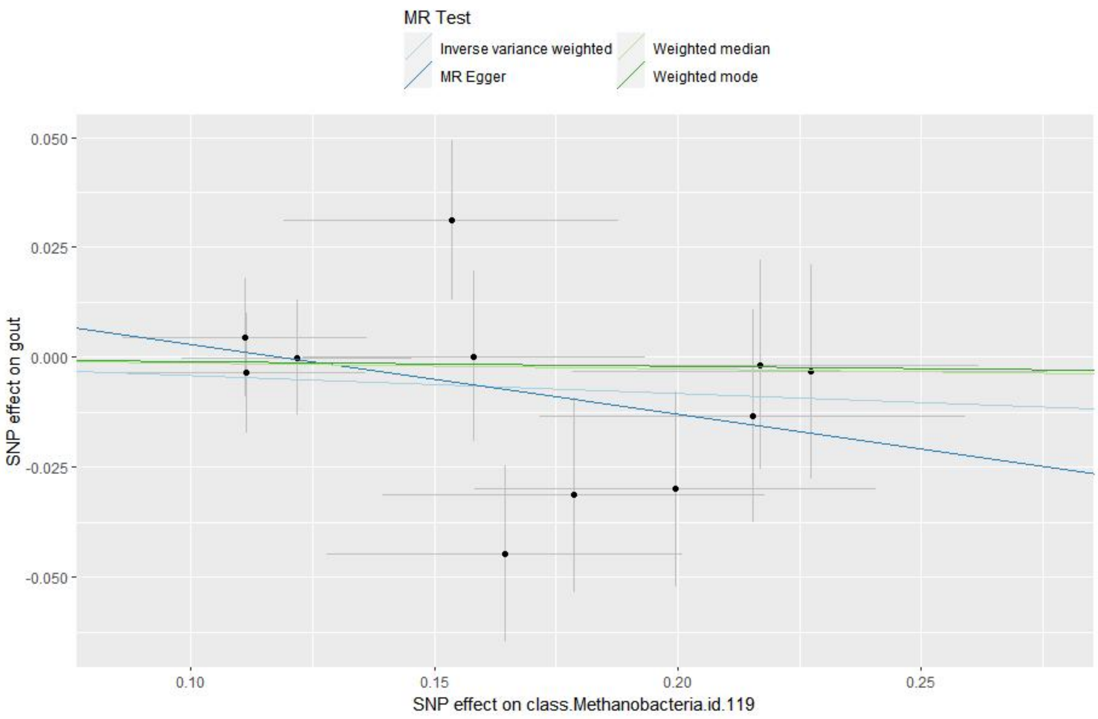

Funnel plot

SUA

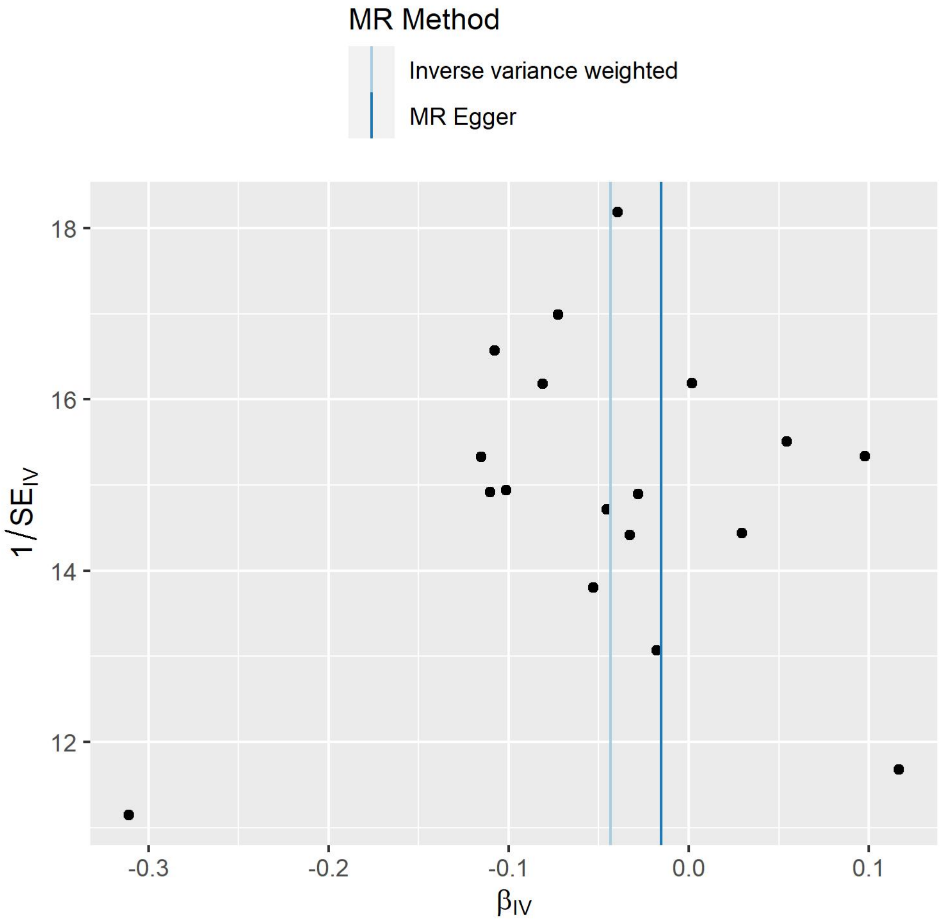

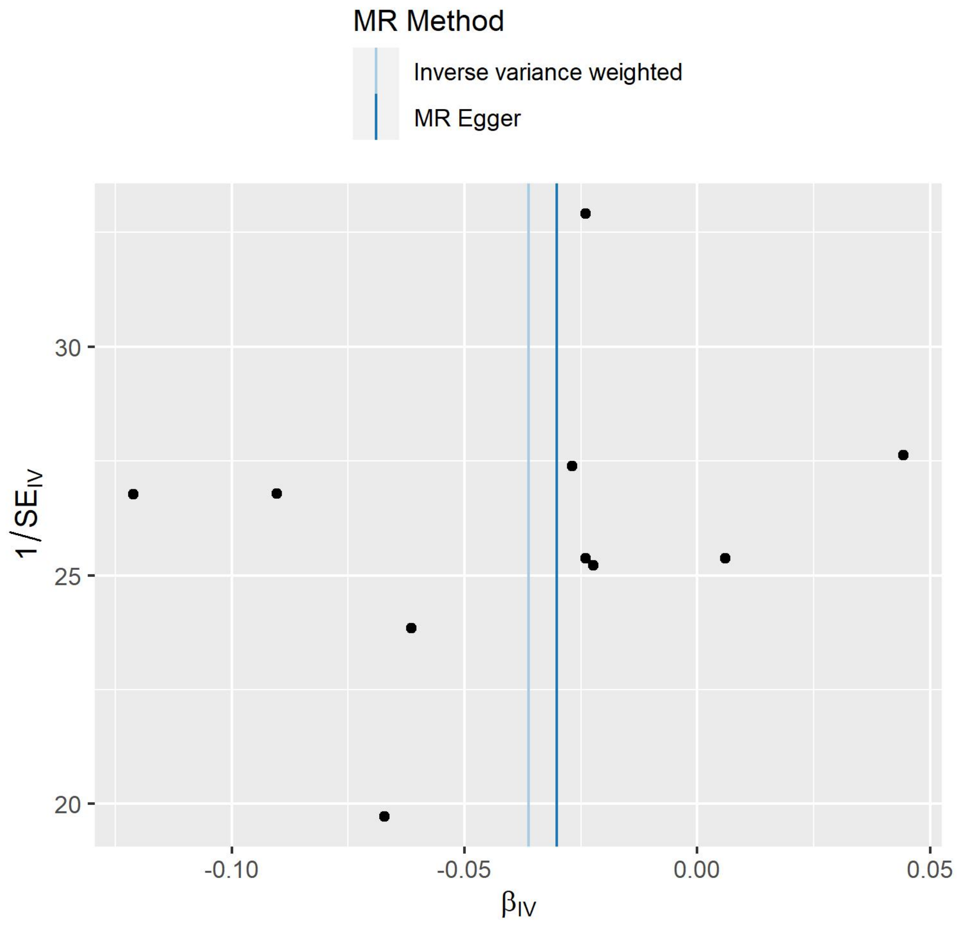
MR Method
Inverse variance weighted
MR Egger
30
25
20
1/SE$_{IV}$
-0.10
-0.05
0.00
0.05
β$_{IV}$

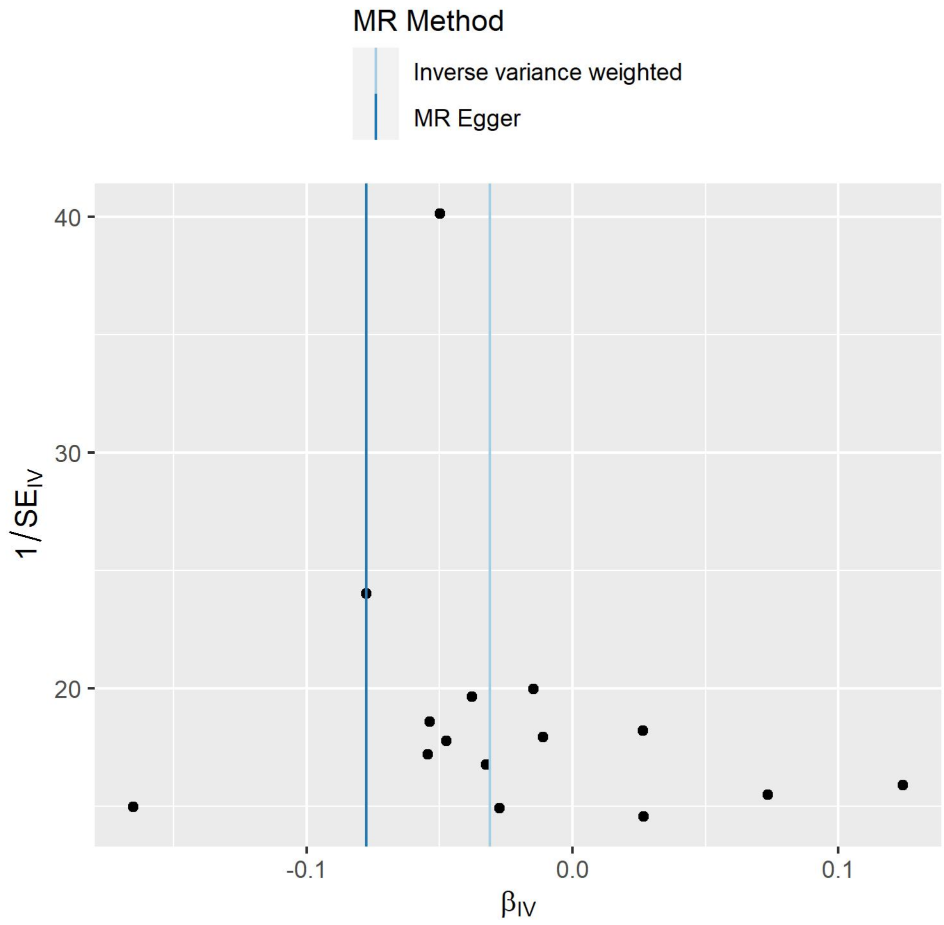

MR Method
Inverse variance weighted
MR Egger
40
30
20
1/SE$_{IV}$
-0.1
0.0
0.1
β$_{IV}$

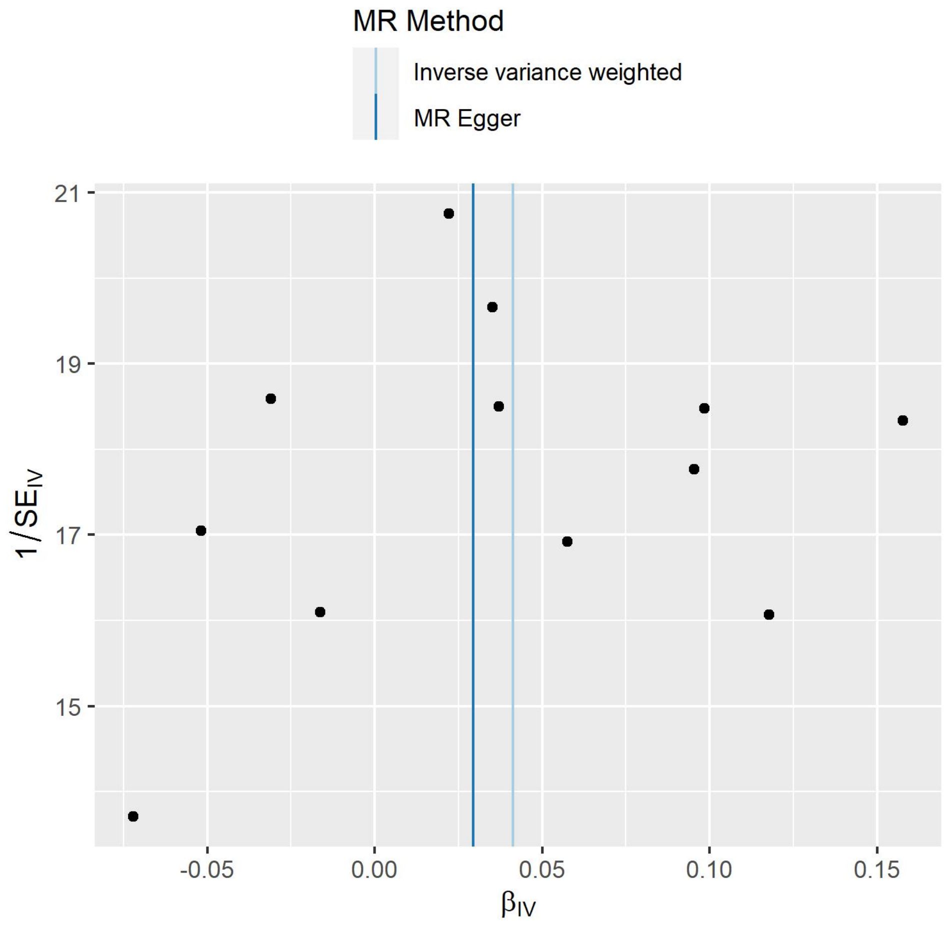
MR Method
Inverse variance weighted
MR Egger
21
19
17
15
$1/SE_{IV}$
-0.05
0.00
0.05
0.10
0.15
β_{IV}

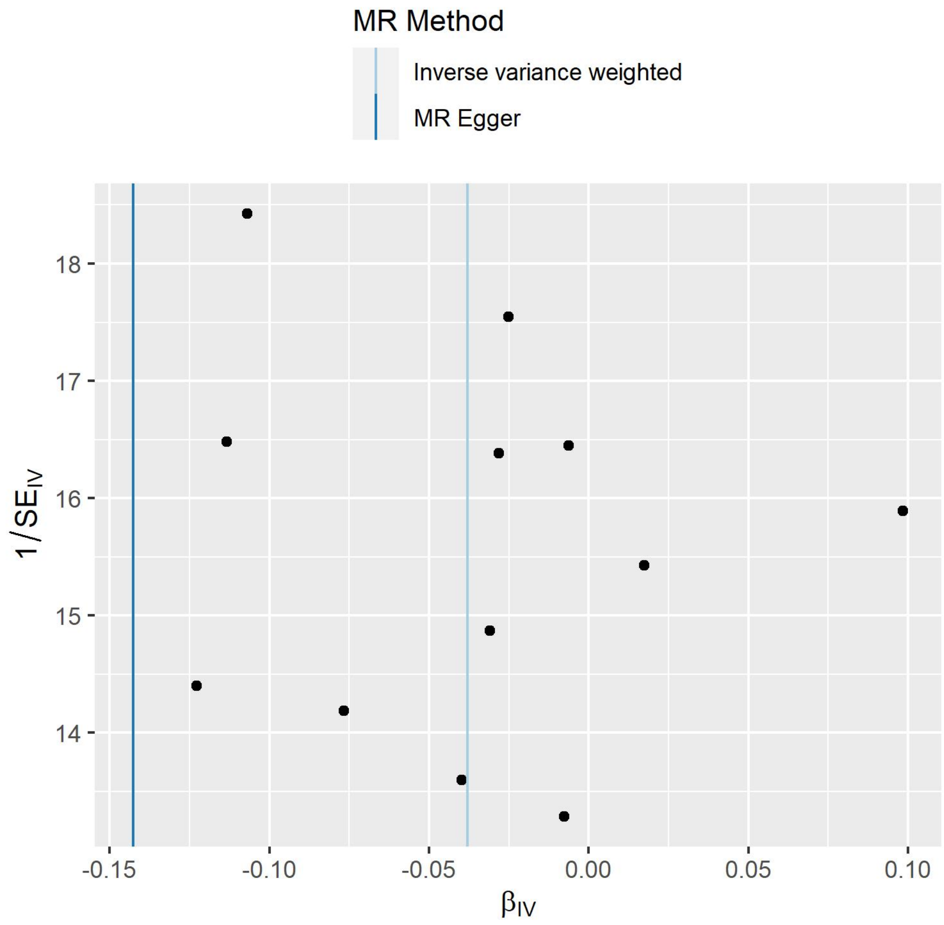
MR Method
Inverse variance weighted
MR Egger
18
17
16
15
14
1/SE$_{IV}$
-0.15
-0.10
-0.05
0.00
0.05
0.10
β$_{IV}$

gout

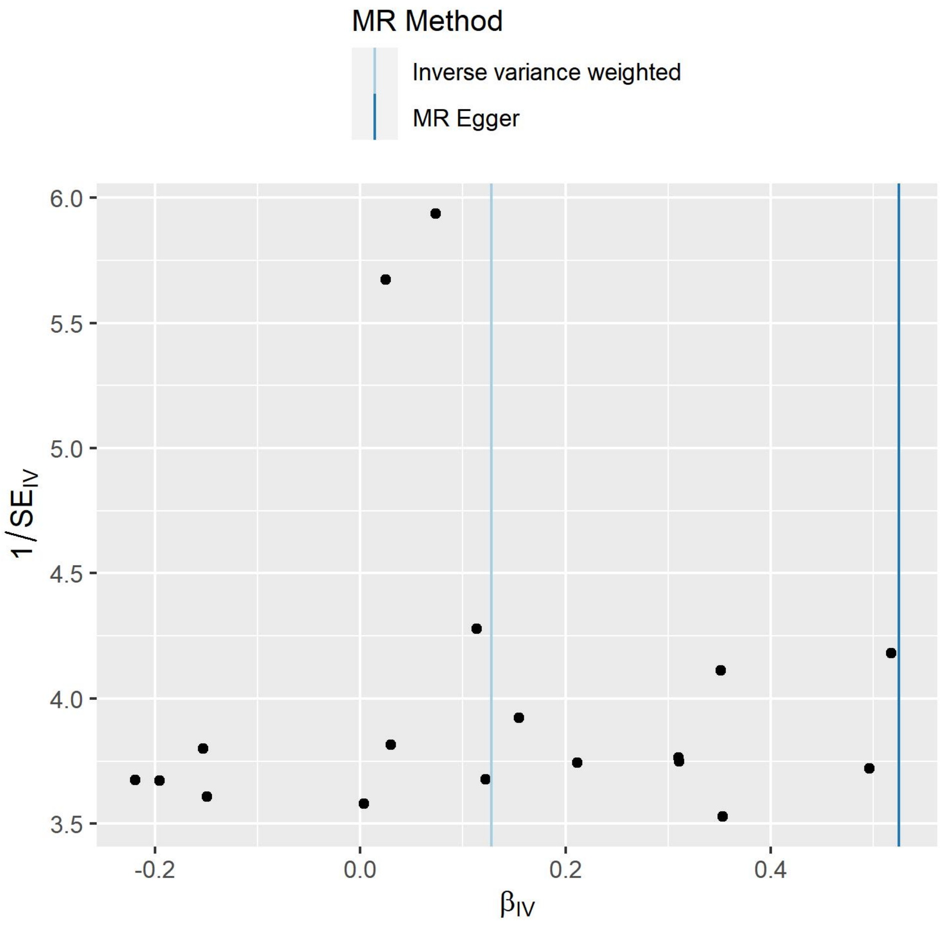

MR Method
Inverse variance weighted
MR Egger
1/SE_IV
β_IV
6.0
5.5
5.0
4.5
4.0
3.5
-0.2
0.0
0.2
0.4

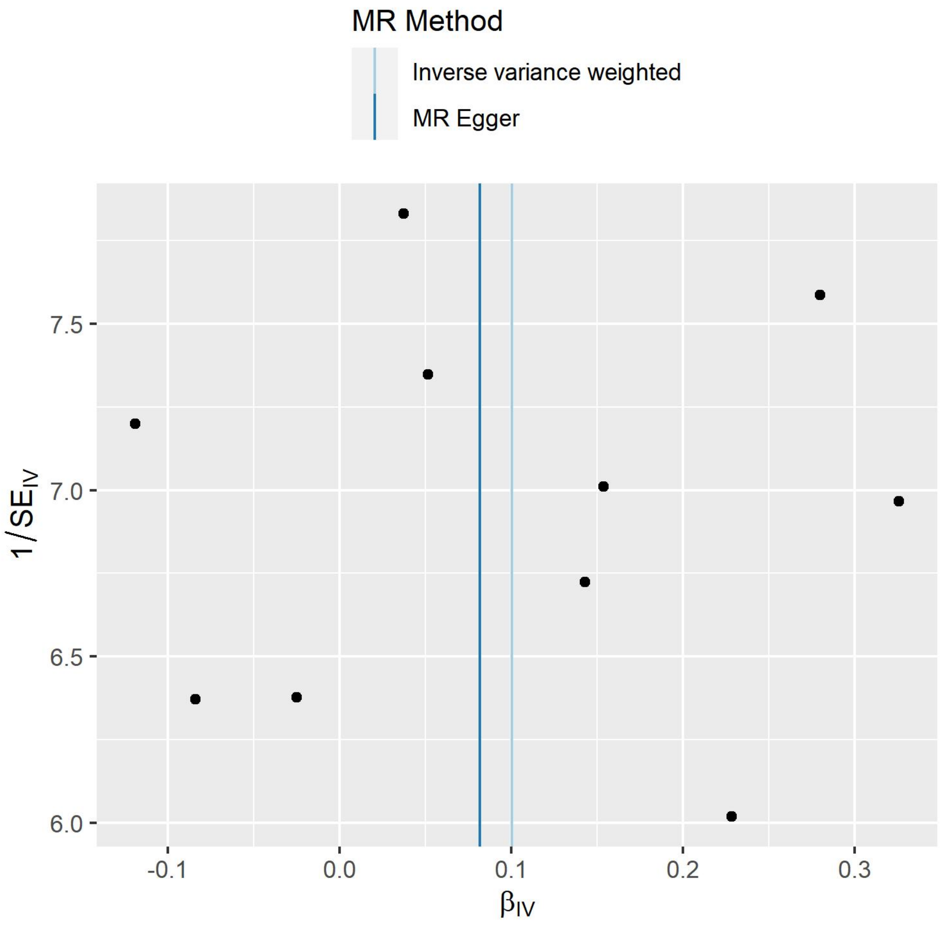
MR Method
Inverse variance weighted
MR Egger
7.5
7.0
6.5
6.0
1/SE_IV
-0.1
0.0
0.1
0.2
0.3
β_IV

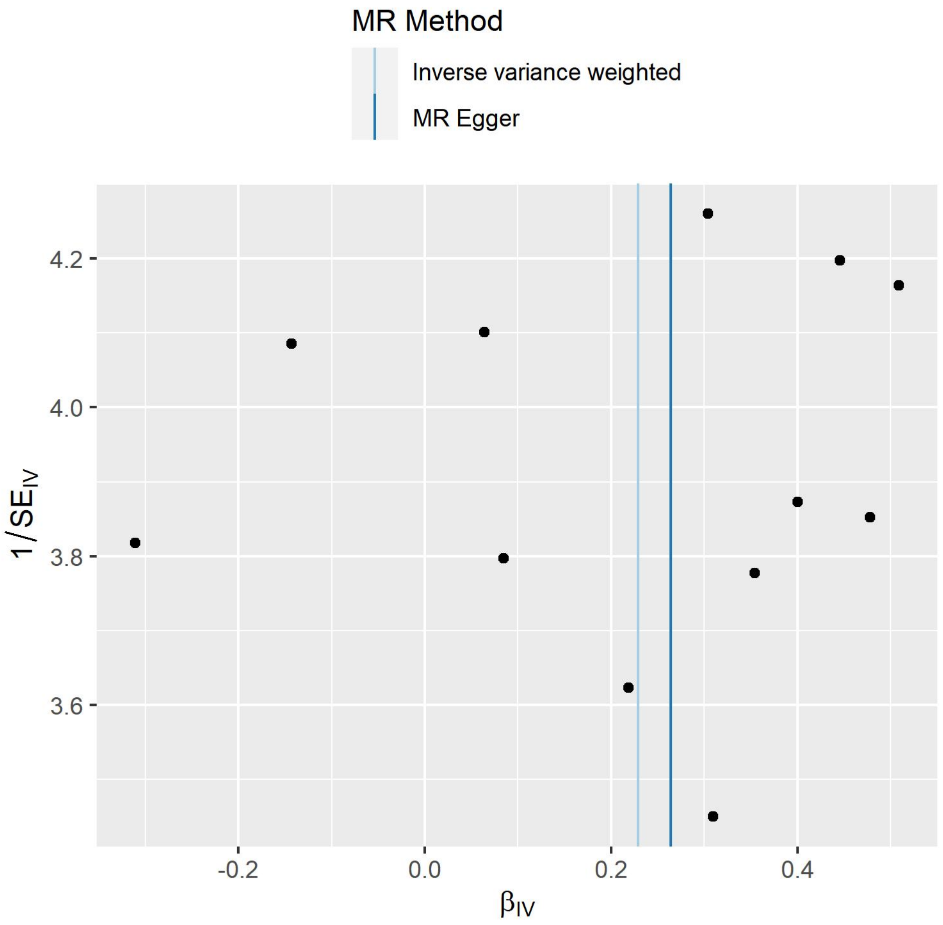

MR Method
Inverse variance weighted
MR Egger
4.2
4.0
3.8
3.6
1/SE_IV
-0.2
0.0
0.2
0.4
β_IV

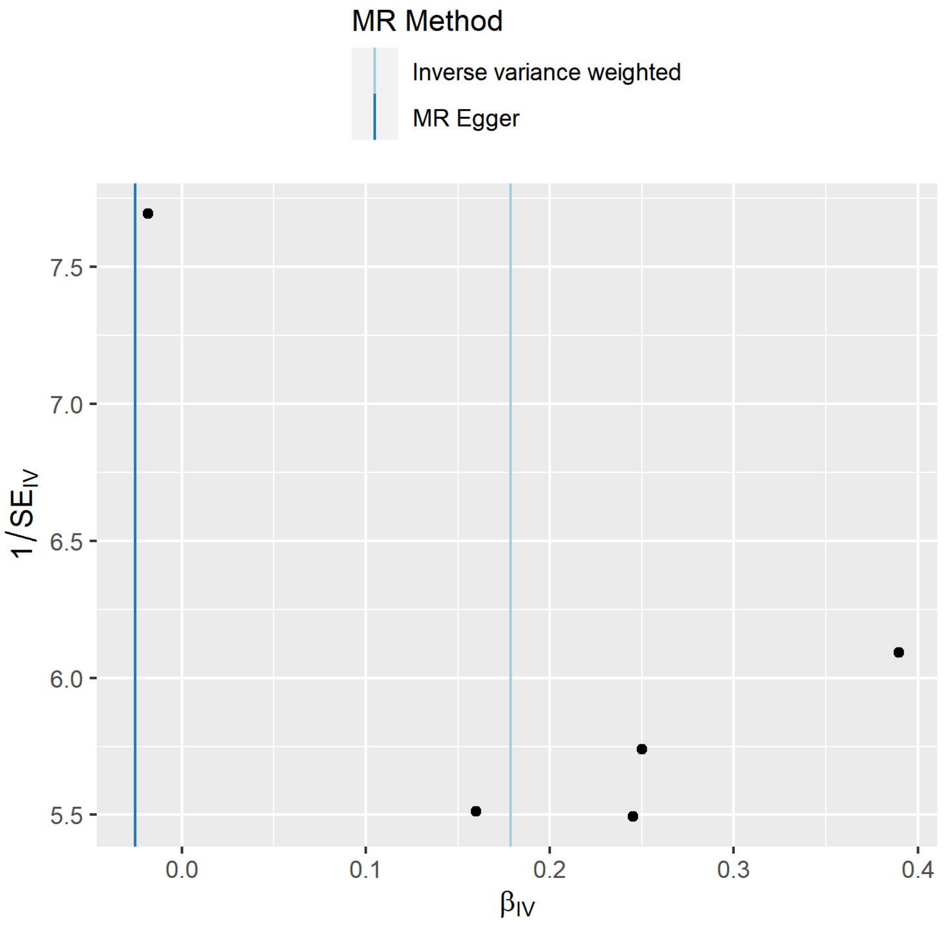
MR Method
Inverse variance weighted
MR Egger
7.5
7.0
6.5
6.0
5.5
1/SE_IV
0.0
0.1
0.2
0.3
0.4
β_IV

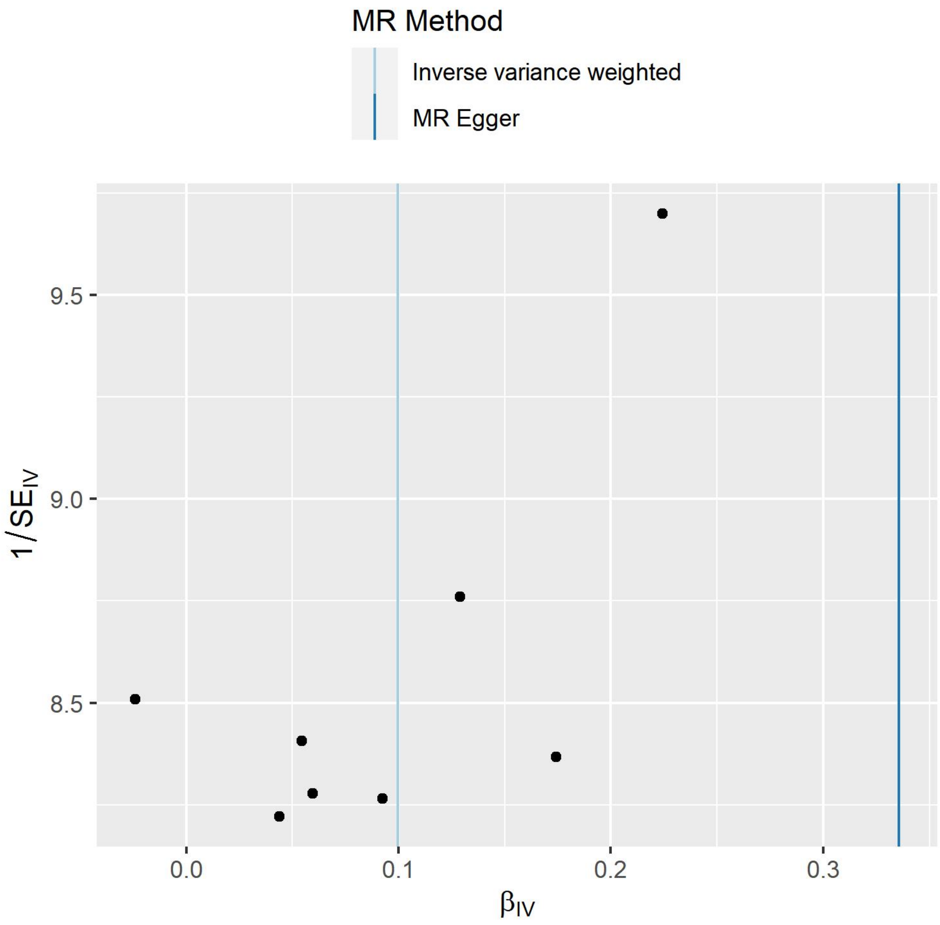
MR Method
Inverse variance weighted
MR Egger
9.5
9.0
8.5
1/SE_IV
0.0
0.1
0.2
0.3
β_IV

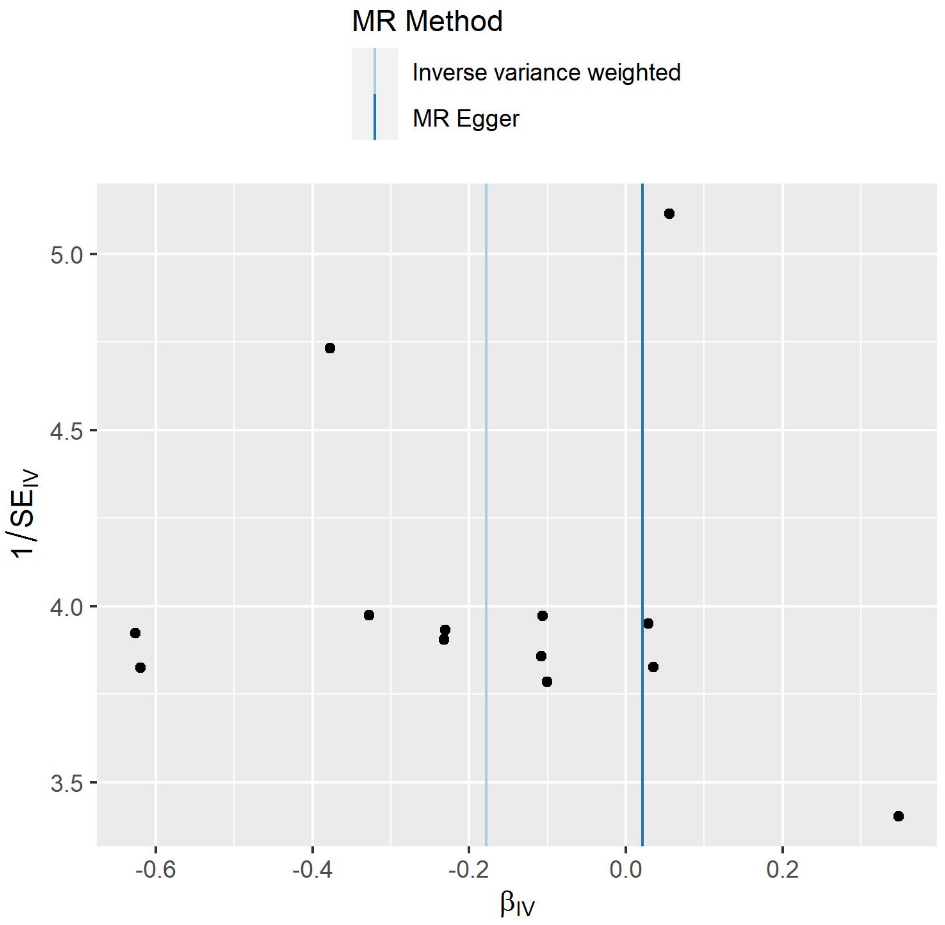
MR Method
Inverse variance weighted
MR Egger
5.0
4.5
4.0
3.5
1/SE$_{IV}$
-0.6
-0.4
-0.2
0.0
0.2
β$_{IV}$

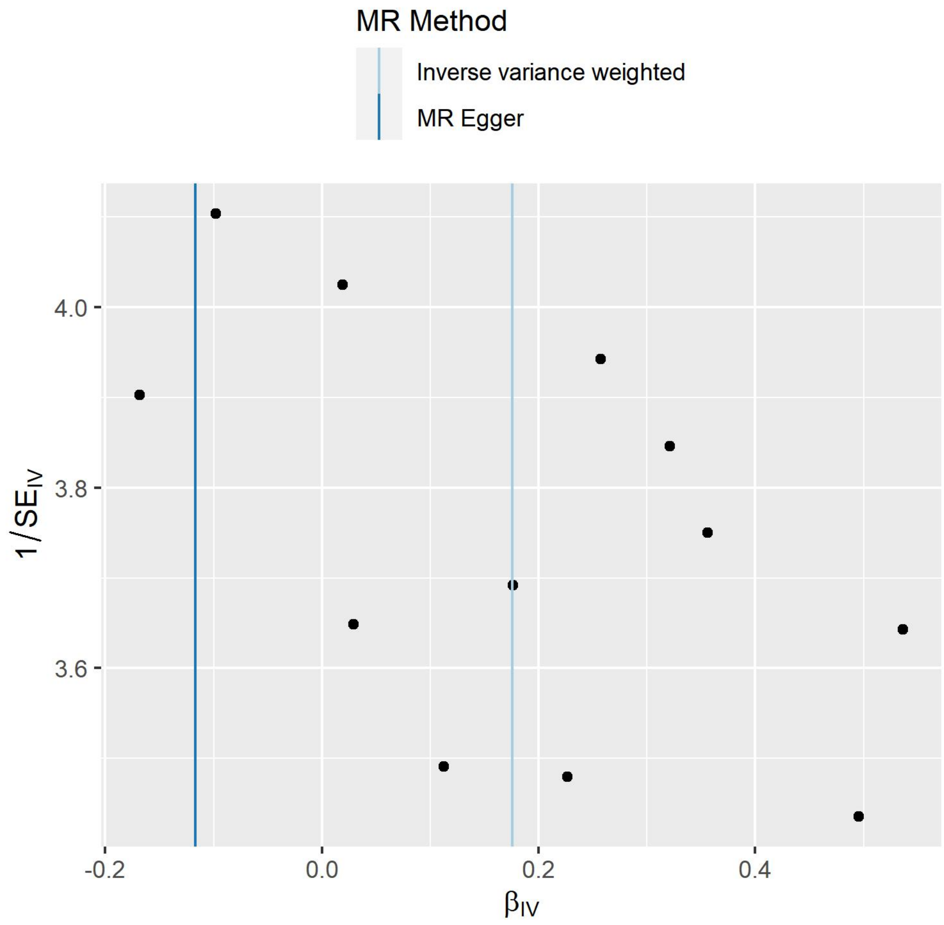
MR Method
Inverse variance weighted
MR Egger
4.0
3.8
3.6
1/SE$_{IV}$
-0.2
0.0
0.2
0.4
β$_{IV}$

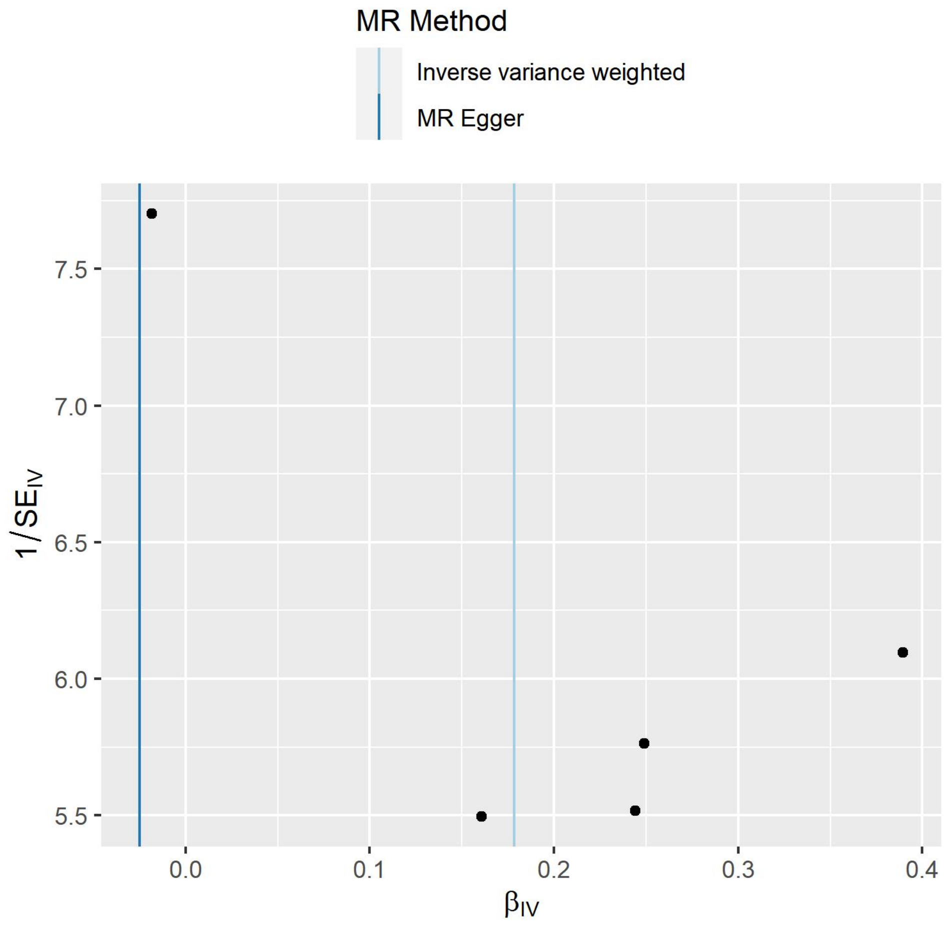

MR Method
Inverse variance weighted
MR Egger
1/SE$_{IV}$
7.5
7.0
6.5
6.0
5.5
0.0
0.1
0.2
0.3
0.4
β$_{IV}$

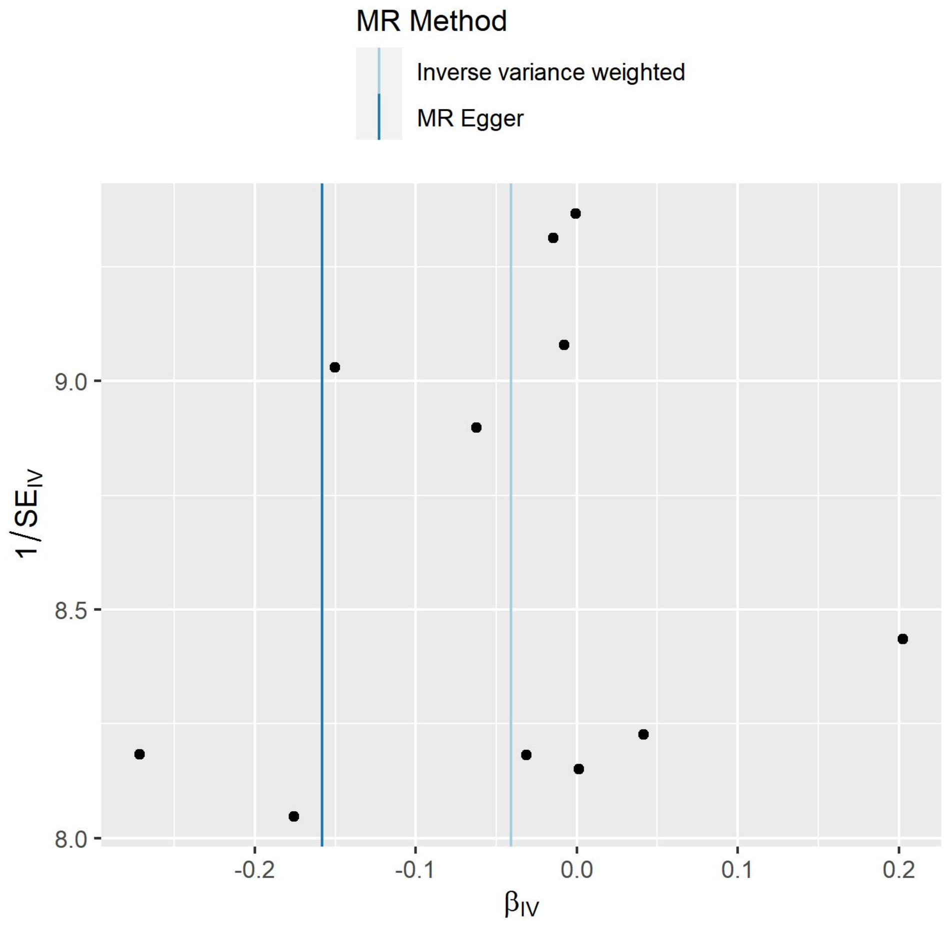
MR Method
Inverse variance weighted
MR Egger
9.0
8.5
8.0
1/SE$_{IV}$
-0.2
-0.1
0.0
0.1
0.2
β$_{IV}$

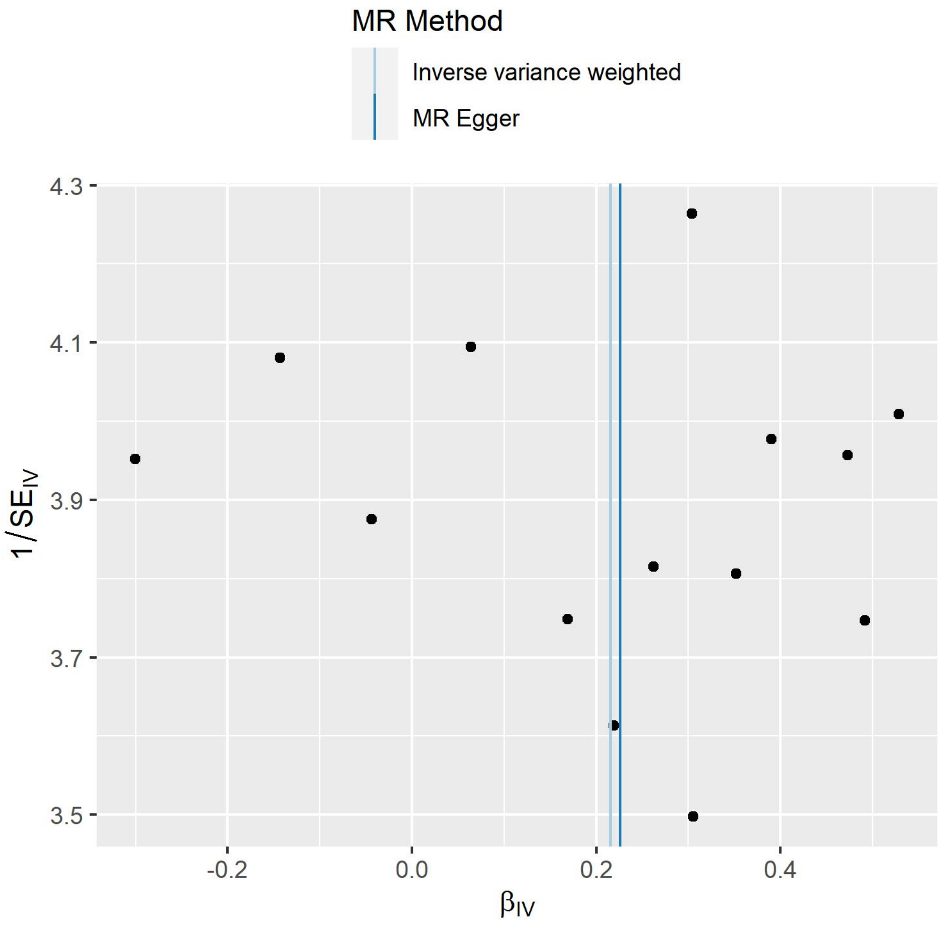
MR Method
Inverse variance weighted
MR Egger
4.3
4.1
3.9
3.7
3.5
1/SE$_{IV}$
-0.2
0.0
0.2
0.4
β_{IV}
