# Supplementary figures and images for: Causal Relationship between Gut Microbiota and Gout: A Two-Sample Mendelian Randomization Study
Source: Nutrients. 2023 Oct 5;15(19):4260. doi: 10.3390/nu15194260 (PMC10574468; doi:10.3390/nu15194260)

Leave-one-out analysis  
SUA

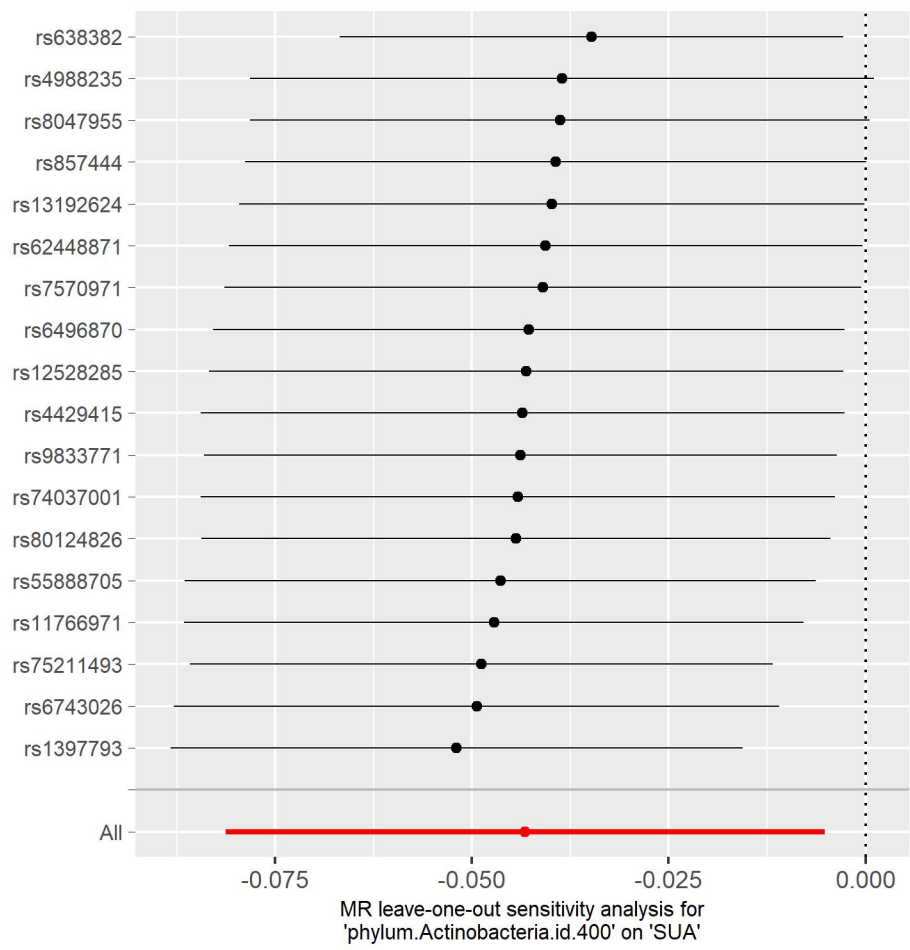

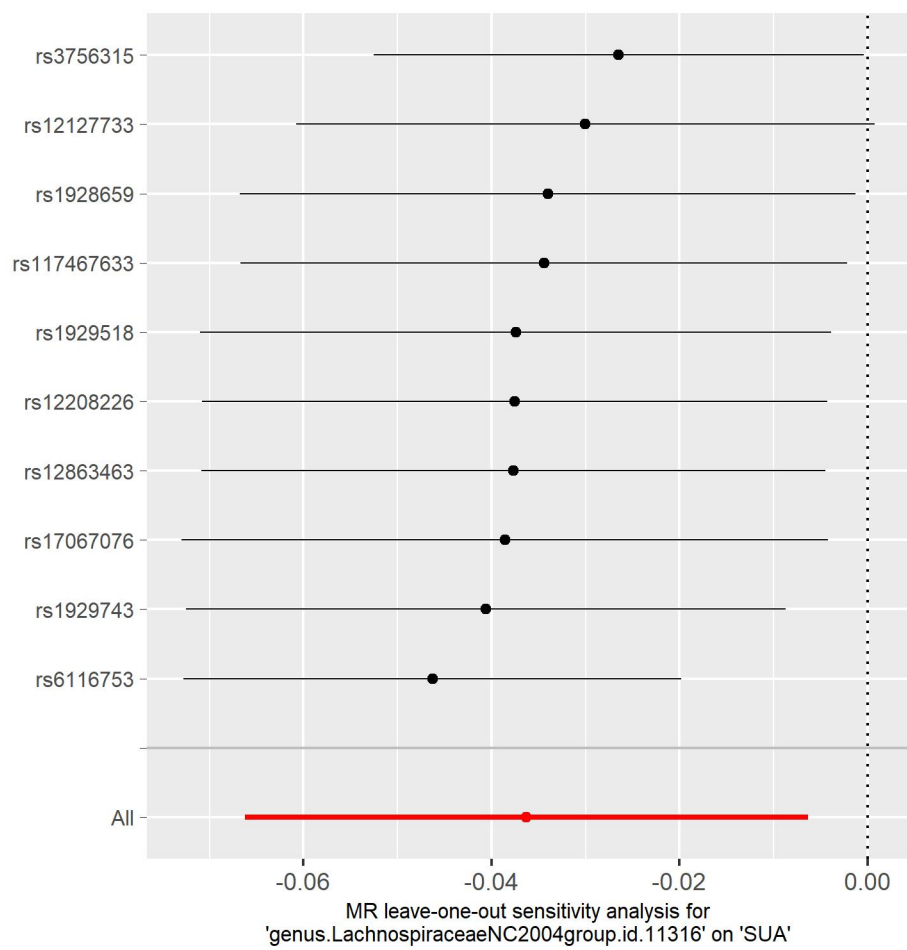

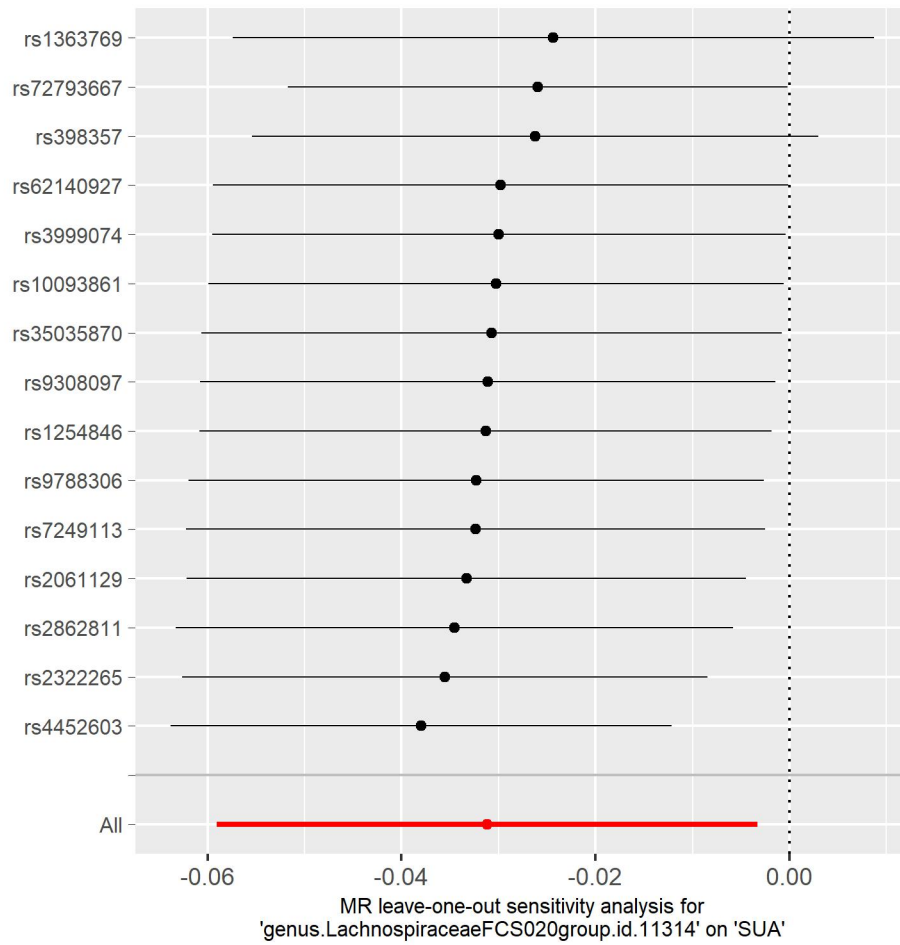

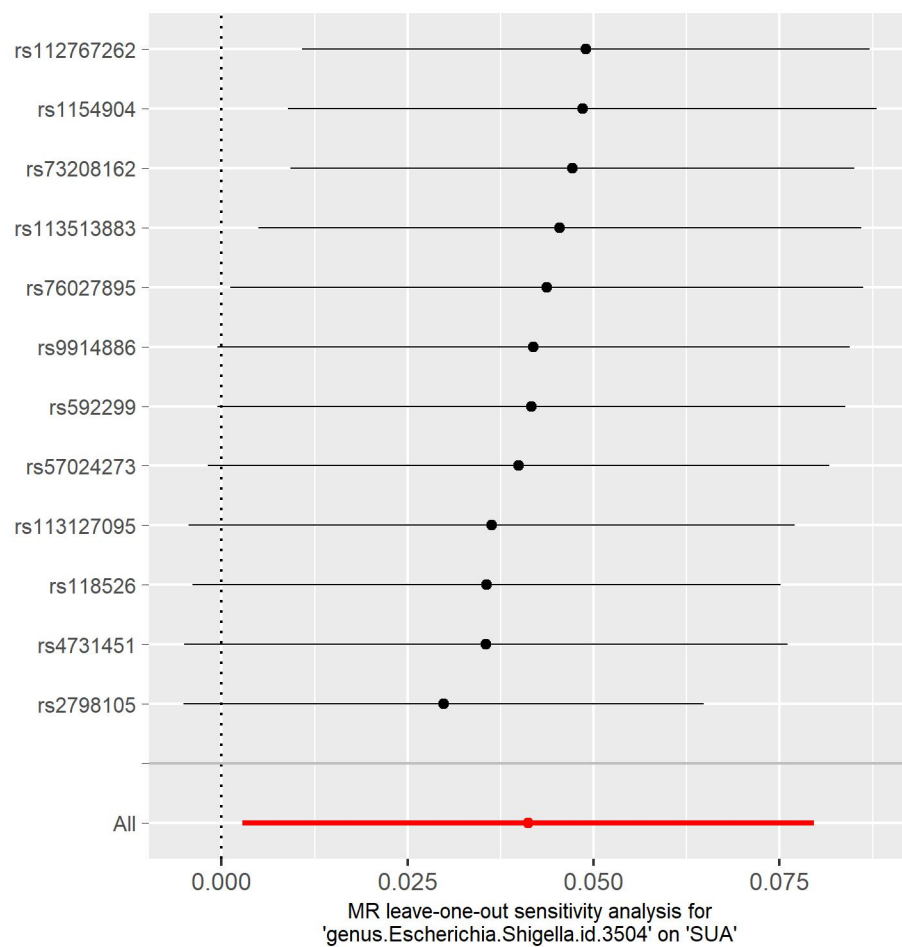

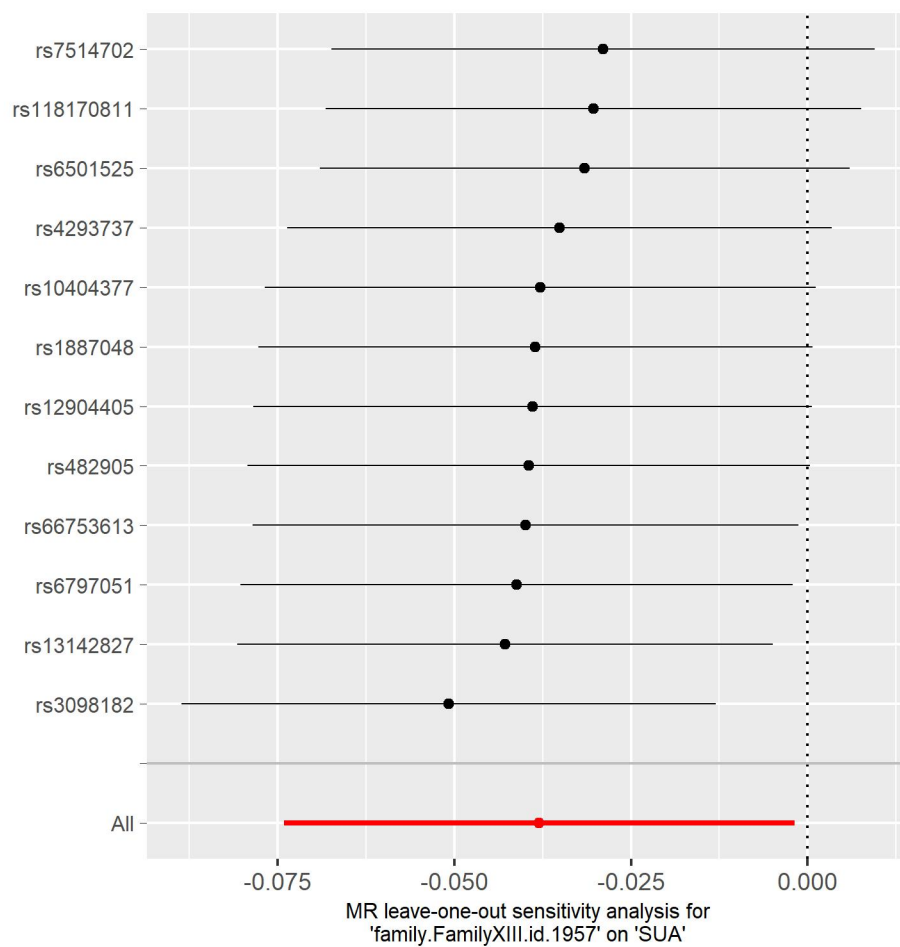

**gout**

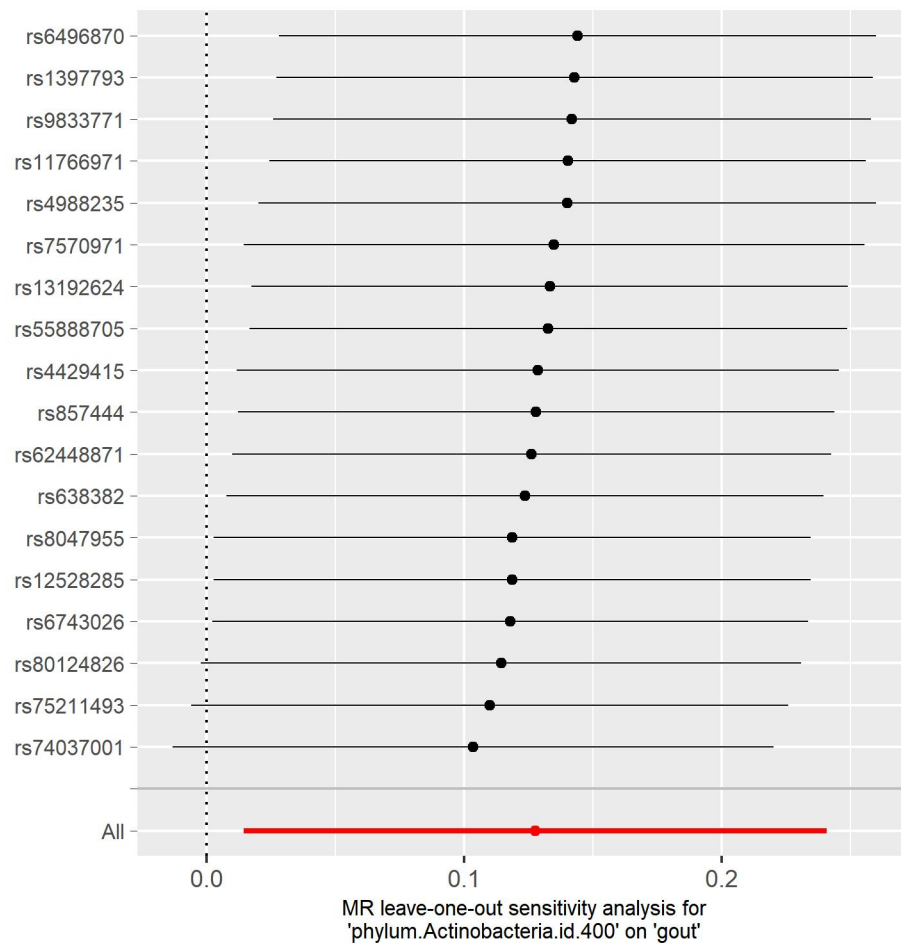

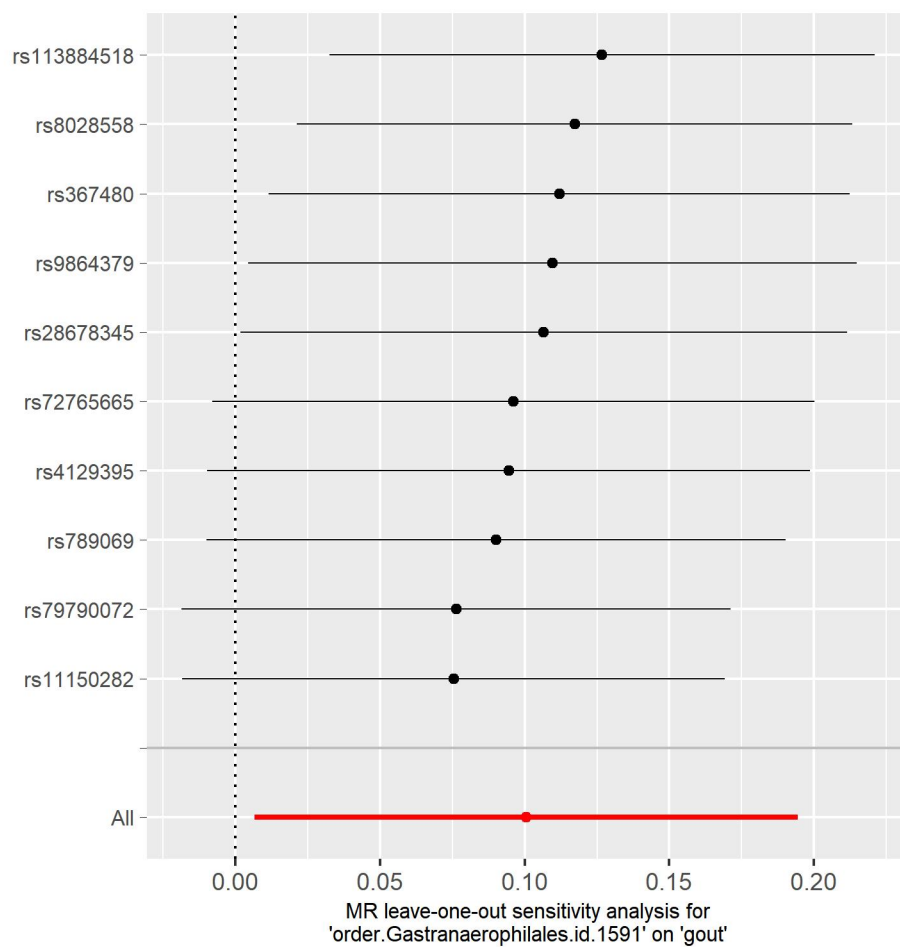

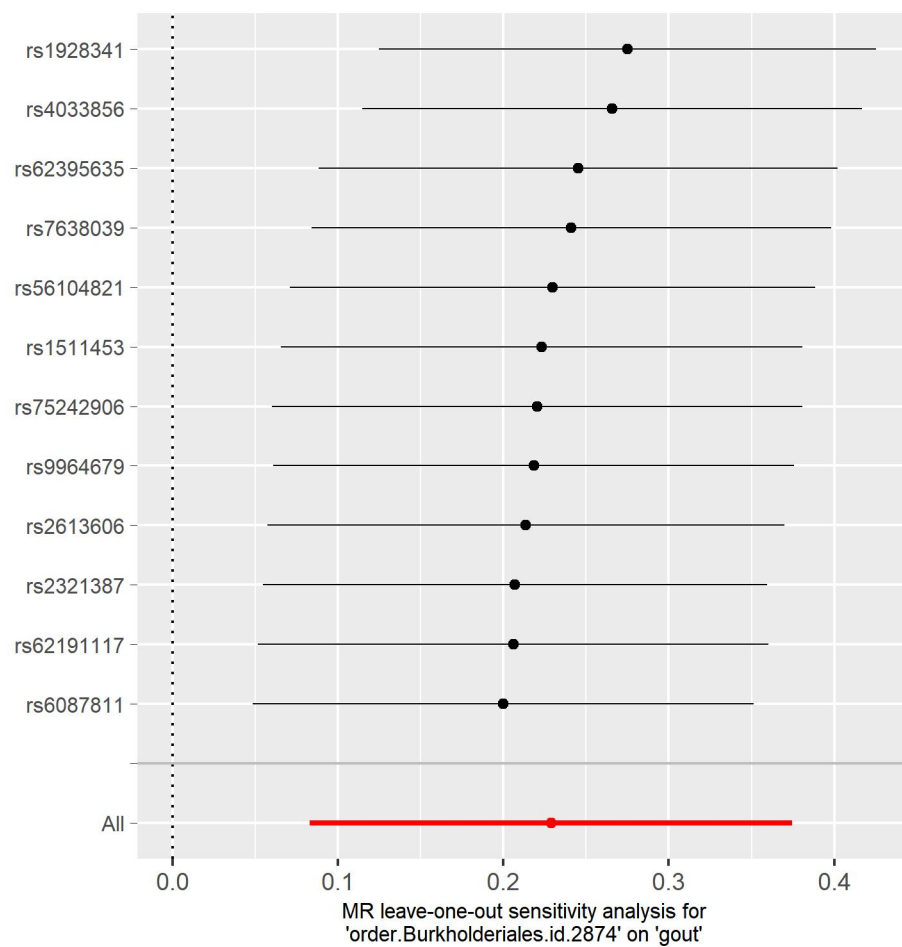

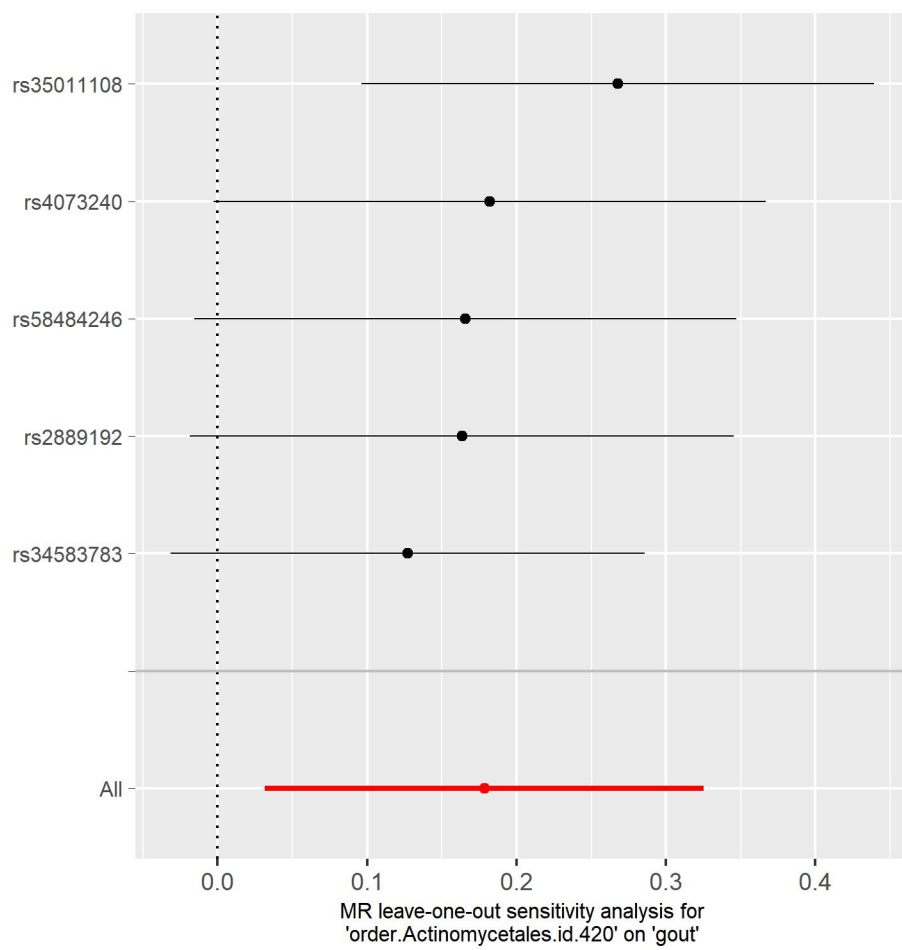

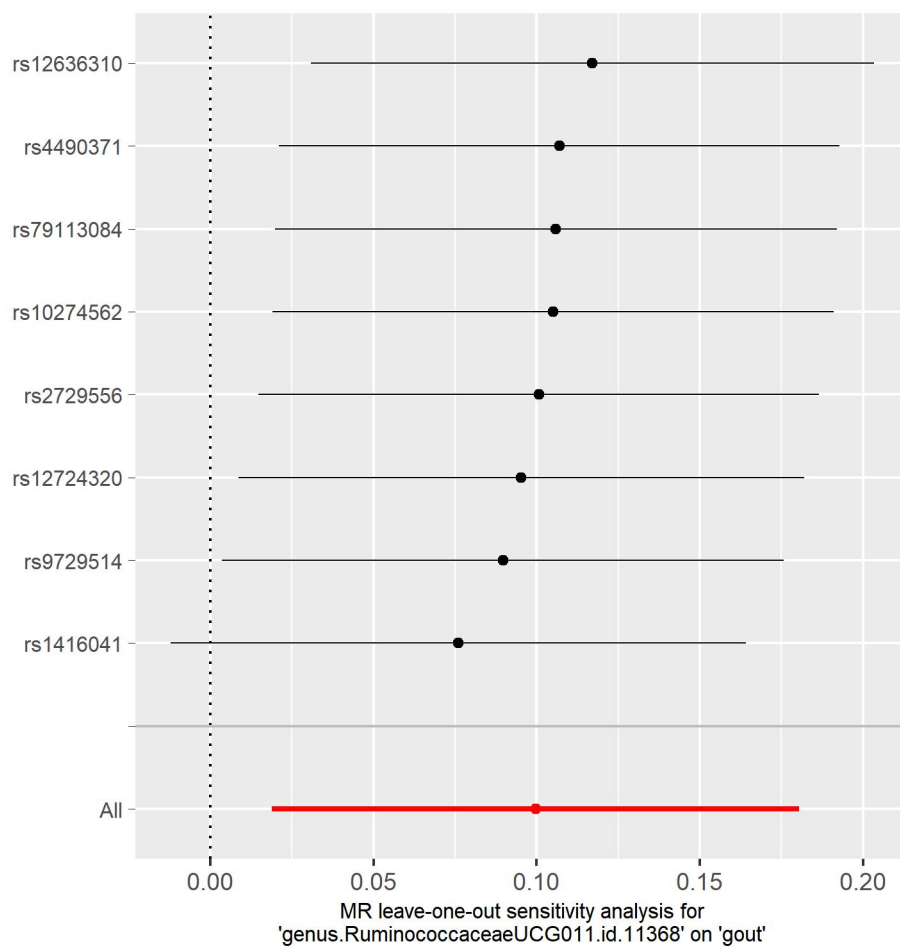

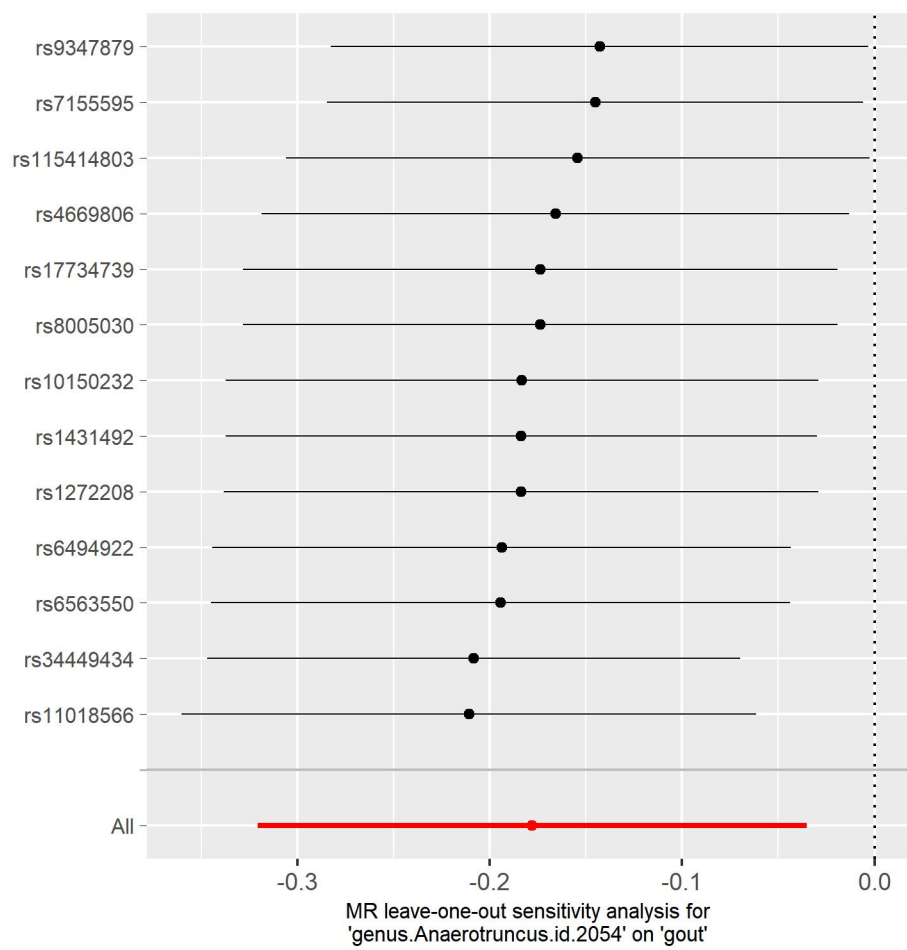

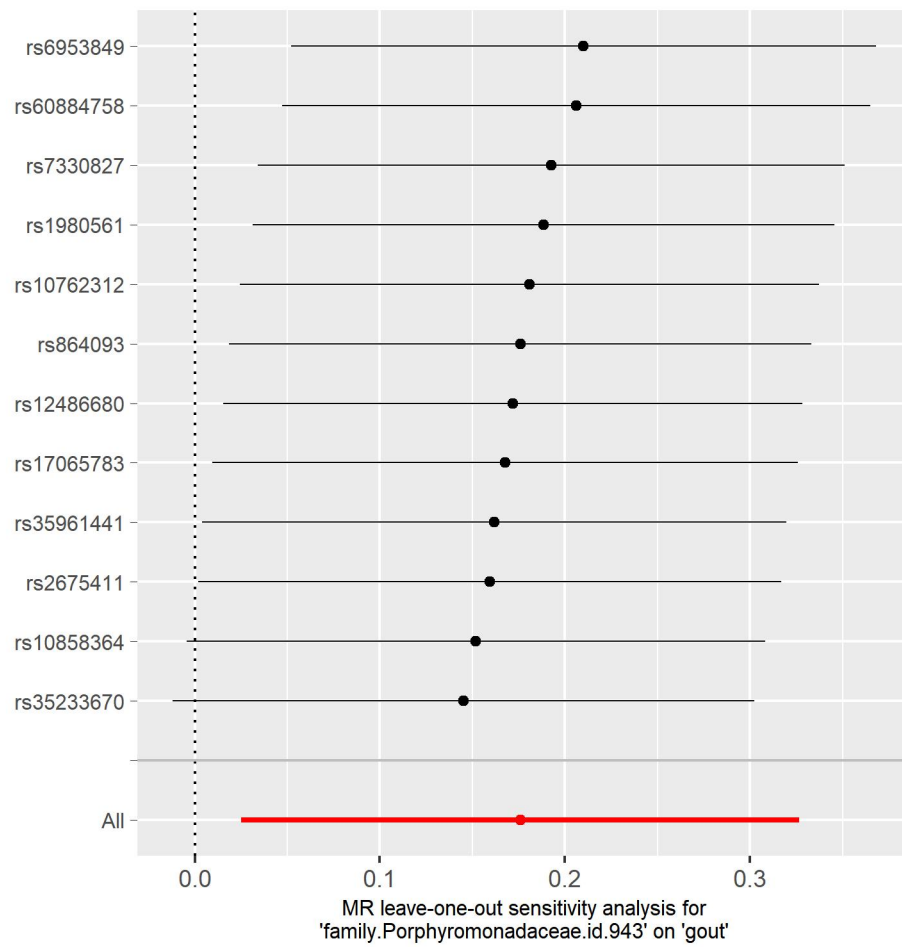

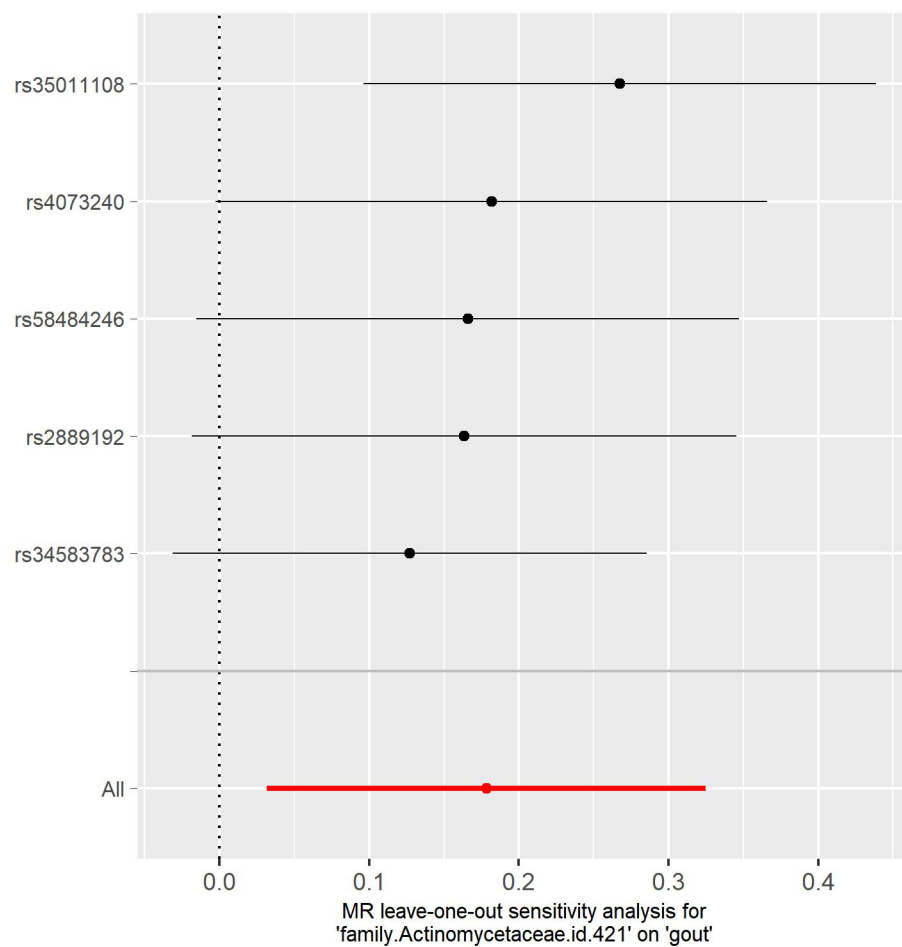

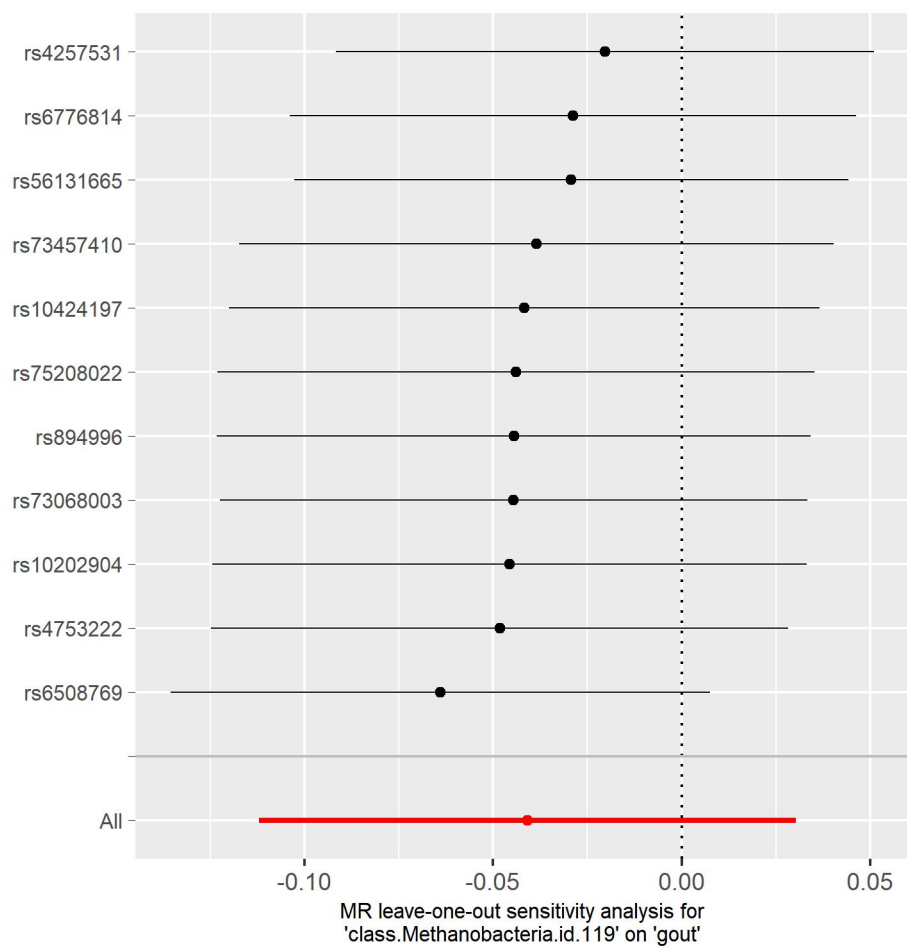

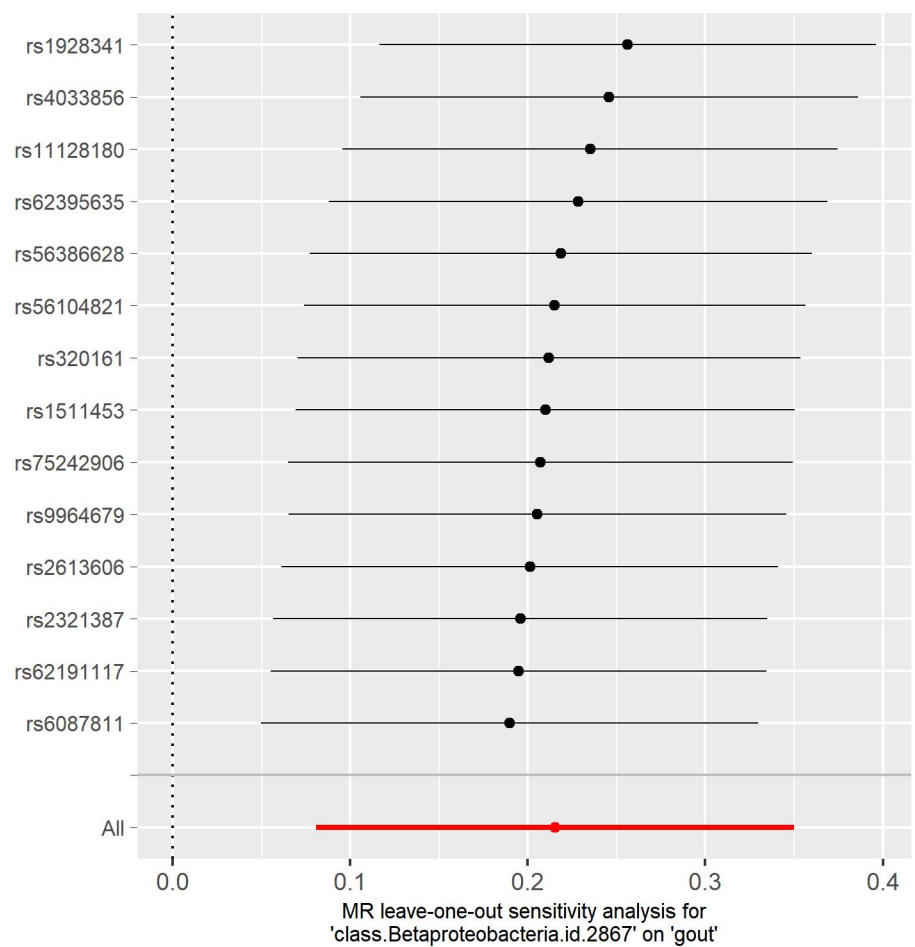

Supplement: Supplementary file 1 [file nutrients-15-04260-s001.zip › Figure_S2.pdf]
